# Stochastic Nanoscale Biophysical Cues as a Basis for the Induction of Glioblastoma‐Like Transcriptional Programs in Astrocytes

**DOI:** 10.1002/advs.202509362

**Published:** 2026-02-03

**Authors:** Laurent Starck, Tobias Butelmann, Sabrina Hogan, Melika Sarem, Bernd Heimrich, Ritwick Sawarkar, Marie‐Françoise Ritz, Gregor Hutter, V. Prasad Shastri

**Affiliations:** ^1^ Institute for Macromolecular Chemistry University of Freiburg Freiburg Germany; ^2^ BIOSS Centre for Biological Signalling Studies University of Freiburg Freiburg Germany; ^3^ Brain Tumor Immunotherapy and Biology Lab Department of Biomedicine University of Basel Basel Switzerland; ^4^ Faculty of Medicine Department of Neuroanatomy University of Freiburg Freiburg Germany; ^5^ Institute for Genetics and Medical Research Council Toxicology Unit University of Cambridge Cambridge UK; ^6^ Department of Neurosurgery University Hospital Basel Basel Switzerland

**Keywords:** activated astrocytes, cancer phenotype, mechanobiology, MMP‐2, p53, senescence, spheroids

## Abstract

Although direct biological factors underlying the progression of Glioblastoma (GBM), an aggressive form of brain cancer, have been extensively studied, emerging evidence suggests that indirect biological triggers, such as traumatic brain injury (TBI), may also have a role. Since proteoglycans, secreted by reactive astrocytes and astroglial cells contribute to biophysical characteristics (stochastic topography, stiffness) of the brain, we postulated a role for stochastic nanoroughness in the induction of glioma following brain trauma. Using a model system to emulate such physical cues that manifest following traumatic injury, we demonstrate that human cortical astrocytes undergo spontaneous organization into spheroids in response to nanoroughness and retain the spheroid phenotype even upon withdrawal of the physical cues. Furthermore, spheroids serve as aggregation foci for naïve astrocytes, express activated MMP2, and disseminate upon implantation in the mouse brain. RNA‐seq analysis revealed that astrocytes within spheroids differentially express genes, including p53, ADAMTS proteases, and NOTCH3, and adopt a transcriptional program enriched for GBM proneural signatures, with reactome analysis pointing toward astrocytes with GBM‐associated transcriptional traits. Moreover, nanoroughness mediates a cross‐talk between cancer cells and astrocytes through induced senescence. These findings implicate a role for stochastic biophysical cues in driving a potential malignant transformation of astrocytes.

## Graphical abstract

Stochastic nanoscale physical cues induce glioblastoma (GBM)‐associated transcriptional traits in naïve astrocytes leading to spontaneous formation of spheroids. Cells within spheroids express activated‐MMP2 and a differential gene expression pattern involving P53 and NOTCH3, providing evidence for a role for changes in brain topography, as observed in TBI, in malignant transformations of astrocytes, and as a potential trigger of GMB.

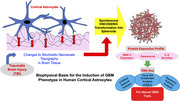

## 1 Introduction

Tumors of the CNS, accounting for approximately 2% of neurological diseases and less than 2% of diagnosed cancers, have become a significant public health concern, with a marked 17% increase in incidence since 1990 [1]. GBM, characterized by its aggressiveness, limited treatment options, and low life expectancy, presents a formidable challenge in the field of neuro‐oncology [2]. While numerous factors contribute to the progression of GBM, including soluble signals and alterations in astrocyte phenotype, recent studies have shed light on the potential role of indirect biological triggers. Notably, it has been suggested that traumatic brain injury (TBI) increases the predisposition to gliomas later in life [3, 4]. While one noteworthy study has concluded an absence of such a correlation between TBI and primary brain cancer [5], reports have also emerged describing GBM formation directly at the site of injury, years following the traumatic event [6, 7], and radiological evidence from several case reports supports this correlation between TBI and glioma [6, 8]. Recently, a case study has reported the occurrence of GBM in the vicinity of TBI in servicemen who suffered head trauma [9]. In a commentary, Ohana et al., employ the term “traumatic glioblastoma” to describe the correlation between TBI and GBM, and argue that p53, hypoxia‐inducible factor‐1a, and c‐MYC, three transcription factors associated with TBI, have also been identified in glioblastoma, thus providing a cause‐and‐effect relationship [10]. Moreover, pediatric brain trauma has been associated with a twofold increase in the likelihood of brain cancer development [11]. Given the strong association between cancer and inflammation [12], it is plausible that trauma‐induced inflammation may promote progenitor cell transformation, potentially contributing to glioma initiation [3, 13]. Immunohistochemical studies have revealed reactive astrocytes (tumor‐associated astrocytes, TAAs) surrounding glioblastoma, which interact closely and communicate with cancer cells by producing proteases, cytokines, and growth factors [14, 15, 16]. Initially, astrocyte reactivity appears to restrict the glioma progression; however, in the later stages of the disease, it ultimately favors invasion [17], suggesting a dual role of astrocytes in initiating and maintaining gliomas [18].

Mechanobiology, the interplay between physical input and biological processes, represents a prominent alternative mechanism for modulating cell behavior [19]. Over the past two decades, a clear picture of the role of mechanobiology in cell phenotype and fate has emerged [20, 21, 22, 23], and changes to matrix stiffness have been linked to cancer progression [24, 25, 26]. Despite the brain's characteristic softness (E modulus 1.3 – 1.9 KPa) [27, 28] and the absence of substantial fibrous extracellular matrix proteins, brain tissue is rich in chondroitin sulfate (CS) and heparin sulfate proteoglycans (PGs) secreted by astrocytes. Proteoglycans can form large aggregates with hyaluronic acid, contribute significantly to the microstructure of the brain [29], and present nanoscale physical cues in the form of stochastic nanoroughness. Previous studies have demonstrated that stochastic nanoroughness impacts stem cell differentiation, morphology, and extracellular matrix assembly [30, 31]. Notably, we have shown that stochastic nanoroughness impacts the lineage commitment of telencephalic neural stem cells and mediates and alters hippocampal neuron and astrocyte behavior via the mechanosensing channel protein Piezo‐1. Our investigations have also revealed altered nanoroughness in Alzheimer's plaques, suggesting a potential role for tissue topography in degenerative processes [32]. More recently, we have demonstrated that glial scarring around neural electrodes can be inhibited and reversed by stochastic roughness, and neural electrodes presenting a defined regimen of stochastic nanoroughness resist glial scarring in vivo through a mechanism involving Piezo‐1 [33]. Blunt insult to the brain often leads to swelling [34] and increased expression of PGs by reactive astrocytes [35], resulting in alterations to the physical space [36] that can impact the mechanical properties of the brain tissue [37]. It has been shown in ovine models that TBI can induce microstructural changes to the brain and weaken its structural integrity, making the subject more prone to brain deformation upon subsequent trauma [38]. TBI has also been shown to impede the clearance of Tau protein via the glymphatic pathway, promoting the formation of Tau protein aggregates, which are granular and 15–25 nm in size [39] and contribute to dementia and degenerative processes in the brain [40]. Tau protein monomer is an intrinsically disordered protein [41], and so are CSPGs. Disordered structures lead to stochastic aggregation [42, 43] and stochastic physical attributes, as organization is not deterministic but random. It has been shown that following TBI, sulfotransferases, which are desulfonation enzymes, are upregulated, leading to changes in the sulfonation pattern of CPSG, and the loss of CPSG that can persist for months [44]. This is accompanied by an increased secretion of other PGs such as neurocan, aggrecan, CSPG4, and lumican [45]. Such changes are bound to alter the stochastic topography of brain tissue around the brain trauma, as demonstrated earlier [32]. Furthermore, it has been shown that synaptic protein expression of ECM proteins, in particular those linked to neurodegenerative conditions such as neurocan and brevican, becomes increasingly stochastic with age [46]. Based on these observations, we hypothesize that the induction of tumor phenotype may have a biophysical basis with a role for stochastic nano‐scale physical cues.

To test this hypothesis, we employed the well‐established stochastic nanoroughness platform [30, 31, 32, 33] and investigated the effect of nano‐scale physical cues on human fetal astrocytes. This model is highly relevant physiologically, as it can emulate the stochastic roughness seen in healthy and pathological human brains, and has been shown to alter the cell plasma membrane distribution of mechanosensing proteins such as Piezo‐1, induced a migratory phenotype and changes to the shape (form factor) in cortical astrocytes [32], and modulate astrocyte behavior in vivo in the superior colliculus region of the brain [33]. Furthermore, our observations that stochastic nanoroughness impacts cell membrane tension and the formation of stress fibers in fibroblasts, the size of punctuate focal adhesion complexes [31], further support its physiological relevance. Our results demonstrate that stochastic physical cues can trigger phenotypic changes in astrocytes, leading to the spontaneous formation of spheroids, which possess many of the transcriptional traits associated with GBM. Furthermore, these spheroids actively stimulate astrocyte migration and induce trafficking of human tumor cells.

## 2 Results

### 2.1 Astrocyte Spheroid Formation Is Triggered by Nanoroughness and Is Independent of Piezo‐1

**FIGURE 1:**
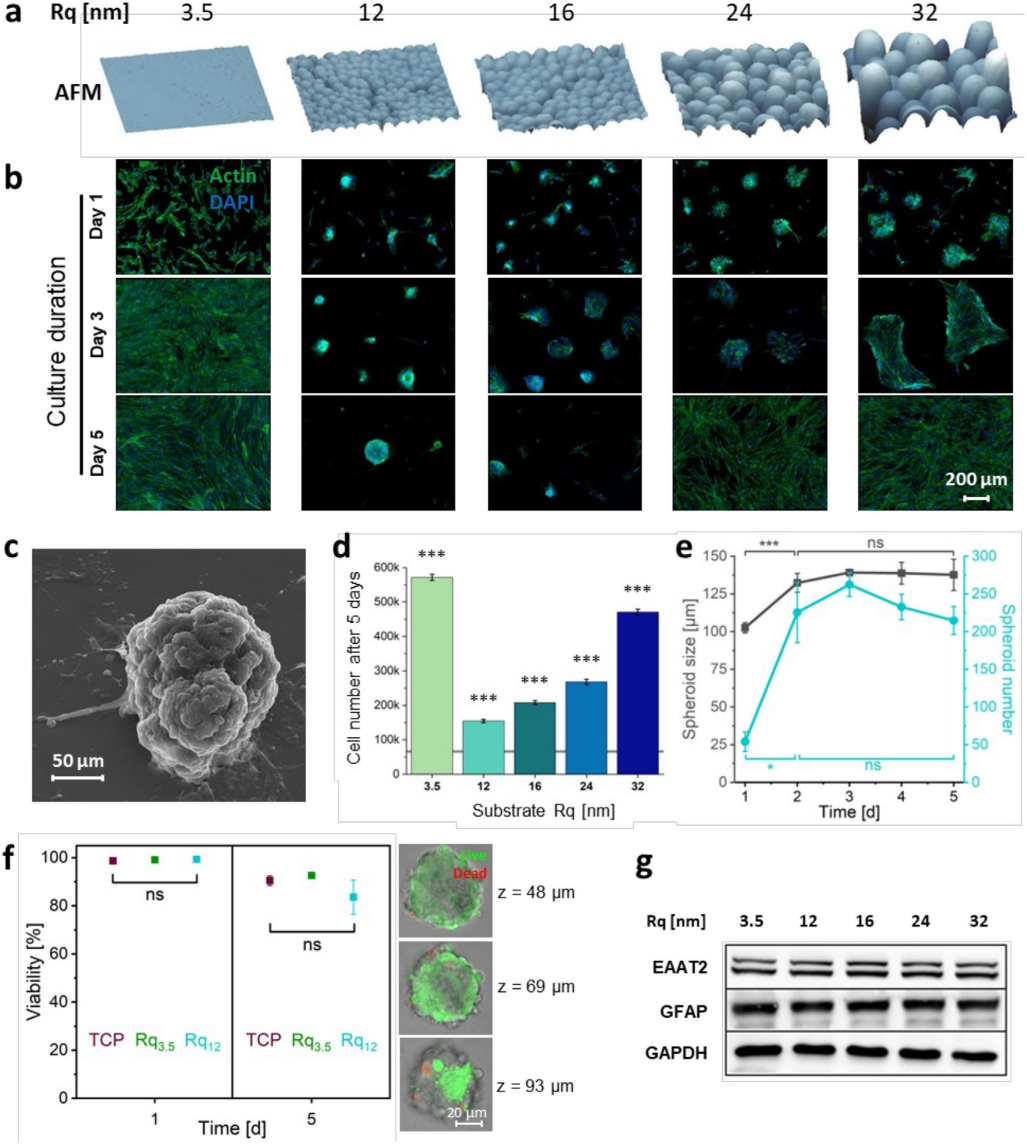
Nanoroughness alters astrocyte phenotype and induces spheroid formation. (a) AFM images of surfaces. (b) Astrocytes grown on varying degrees of nanoroughness show altered morphology revealed using actin staining. (c) SEM picture of astrocytes grown on Rq_12_ for 5 days, which aggregated into 3D spheroids. (d) Cell numbers on each substrate after 5 days of culture, obtained by DNA quantification (n = 3, all groups were statistically different from the initial seeding density shown by the horizontal line (ANOVA applied with the Tukey test ***p < 0.001), and (e) plot of spheroid numbers on the entire substrate as a function of days in culture, and spheroid size on Rq_12_ over 5 days of culture (n = 3, ns = not significant, *p < 0.05, ***p < 0.001). (f) (Left panel), quantification of viable astrocytes on tissue culture plastic (TCP), Rq_3.5_ and Rq_12_ (ns = not significant), after live/dead staining, (right panel), fluorescence confocal microscopy image of an astrocyte spheroid stained after live/dead staining after 5 days of culture on Rq_12_ at three different z‐planes, showing the absence of a necrotic core. Live and dead cells are stained in green and red, respectively. (g) Western blot expression profile of GFAP and EAAT2 in astrocytes exposed to various stochastic nanoroughness. GAPDH served as the internal housekeeping control. The entire gel and western blot for the proteins are shown in Supplementary Figure S5.

The fate of primary human cortical astrocytes on substrates presenting stochastic nanoroughness (Rq) ranging from 12 nm to 32 nm (Figure 1a) over 5 days was investigated. The entire workflow for generating the substrates and the cell seeding is schematically described in Supplementary Figure S1. While astrocytes on the smooth substrate (Rq_3.5_) showed the classical in vitro astrocyte morphology, on substrates with an Rq of 12 nm, a spontaneous condensation of astrocytes was observed, with progressively increasing Rq's yielding less condensed structures (Figure 1b). However, on day 3 and beyond, while the astrocytes on Rq_16_, Rq_24,_ and Rq_32_ nm progressively dissociated to single cells, the spheroid on Rq_12_ retained its 3D‐organization and further matured (Figure 1b). After 5 days of culture, the spheroids formed on Rq_12_ were further characterized using scanning electron microscopy (SEM), providing evidence for a 3D structure composed of aggregated cells (Figure 1c). Coating the substrate with poly‐ornithine, a positively charged synthetic polymer analogous to poly‐L‐lysine, routinely used to support neuronal cell culture, did not affect spheroid formation (Supplementary Figure S2). Enhancing cell‐substrate adhesion using laminin also supported spheroid formation, but some spheroid dissociation was observed by day 5, and likewise, coating with a mixture of laminin and poly‐ornithine yielded similar outcomes (Supplementary Figure S2). Masking the surface with a coating of Geltrex, a commercially available basement membrane matrix mixture, abrogated spheroid formation on Rq_12_ surface and yielded monolayers of astrocytes at day 5, regardless of the substrate, highlighting the importance of physical stochastic cues in the spontaneous spheroid formation observed in astrocytes. In previous studies we have shown that neural electrodes coated with NPs are stable over a 4‐week period at 37°C, and can withstand repeated insertion and withdrawal from agarose gels [33]. Since the substrate surface was stable for the duration of the study, as verified using SEM, and AFM imaging of the Rq_12_ substrate which showed no changes over the initial 24‐h period when the spheroids begin to form (Supplementary Figure S3), the source of the trigger for spheroid formation can be attributed to the physical cues provided by stochastic nanoroughness of the Rq_12_ surface. To probe the role of cell proliferation in the observed phenomenon, cell numbers were quantified at day 5. Although astrocytes proliferated on all surfaces to different degrees, cells on Rq_12_ showed the least proliferation, with increasing Rq correlating with increased proliferation, revealing a direct connection between stochastic nanoroughness and the proliferative status of astrocytes (Figure 1d). Since the seeding density was the same, and their attachment is similar aon glass (Rq_3.5_) and Rq_12_ (Supplementary Figure S4a), this hints at a possible role for the diminished proliferative status of astrocytes on Rq_12_ in the formation of spheroids.

To investigate the development of the spheroids formed on Rq_12_, the number and size were analyzed over time. From day 1 to day 2, the number of spheroids across the entire substrate increased by more than fourfold from an average of 50 per substrate at 24 h, to above 100 per substrate at 48 h, and reaching a peak around 72 h, before consolidation of the spheroids to an average between 200–250 per substrate at 5 days. The average spheroid size increased by more than 25% from an average of 100 µm after 24 h to 125 – 150 µm at 48 h, beyond which it plateaued (Figure 1e, Supplementary Figure S4b), indicating the formation of spheroids on Rq_12_ occurs through migration of the astrocytes and not due to proliferation, as further illustrated in the next section. The proliferation of cells is generally assumed to be lower in a spheroid than in a monolayer culture. Live/dead staining was used to quantify viable astrocytes. This revealed that astrocytes have similar viability (percent live in total population) in the spheroids as on tissue culture plastic (TCP) and Rq_3.5_ (Figure 1f, graphs). Scanning confocal microscopy confirmed the absence of a necrotic core at the center of the spheroids (Figure 1f, right panel). These results are consistent with earlier studies that showed the absence of any oxygen gradient in spheroids even at very high volumetric cell concentration [20]. Taken together, these findings demonstrate that astrocytes precisely sense and respond to stochastic nanoroughness, and this influences their behavior and morphology. Besides the observed morphological changes, the expression of EAAT2, which is responsible for 90% of the glutamate uptake in the adult CNS [47], and GFAP, an intermediate filament protein considered a marker of astrocyte reactivity, were investigated (Figure 1g, Supplementary Figure S5). The expression of EAAT2 and GFAP showed no differences compared to controls, implying that the astrocytes exposed to nanoroughness retain prominent physiological traits. To gain some mechanistic insights into how astrocytes perceive the underlying nano‐scale physical cues, the overall expression patterns of the stretch‐activated ion channel Piezo‐1, which was previously demonstrated to be involved in sensing of stochastic nanoroughness by neurons [32], and integrin αVβ3, because of its widespread expression and implication in many physiological processes and diseases, were probed. Neither GsMTx4, a potent inhibitor of Piezo‐1, known to reversibly inhibit more than 80% of the mechanically induced currents [48], nor blocking antibody toward αVβ3 [49, 50] had any effect on spheroid formation (Supplementary Figure S6), suggesting that astrocytes perceive nano‐scale cues through a mechanism different from neurons.

### 2.2 Astrocyte Spheroids Remain Stable Even Upon Removal of Physical Cues and Serve as Aggregation Centers for Naïve Astrocytes

**FIGURE 2:**
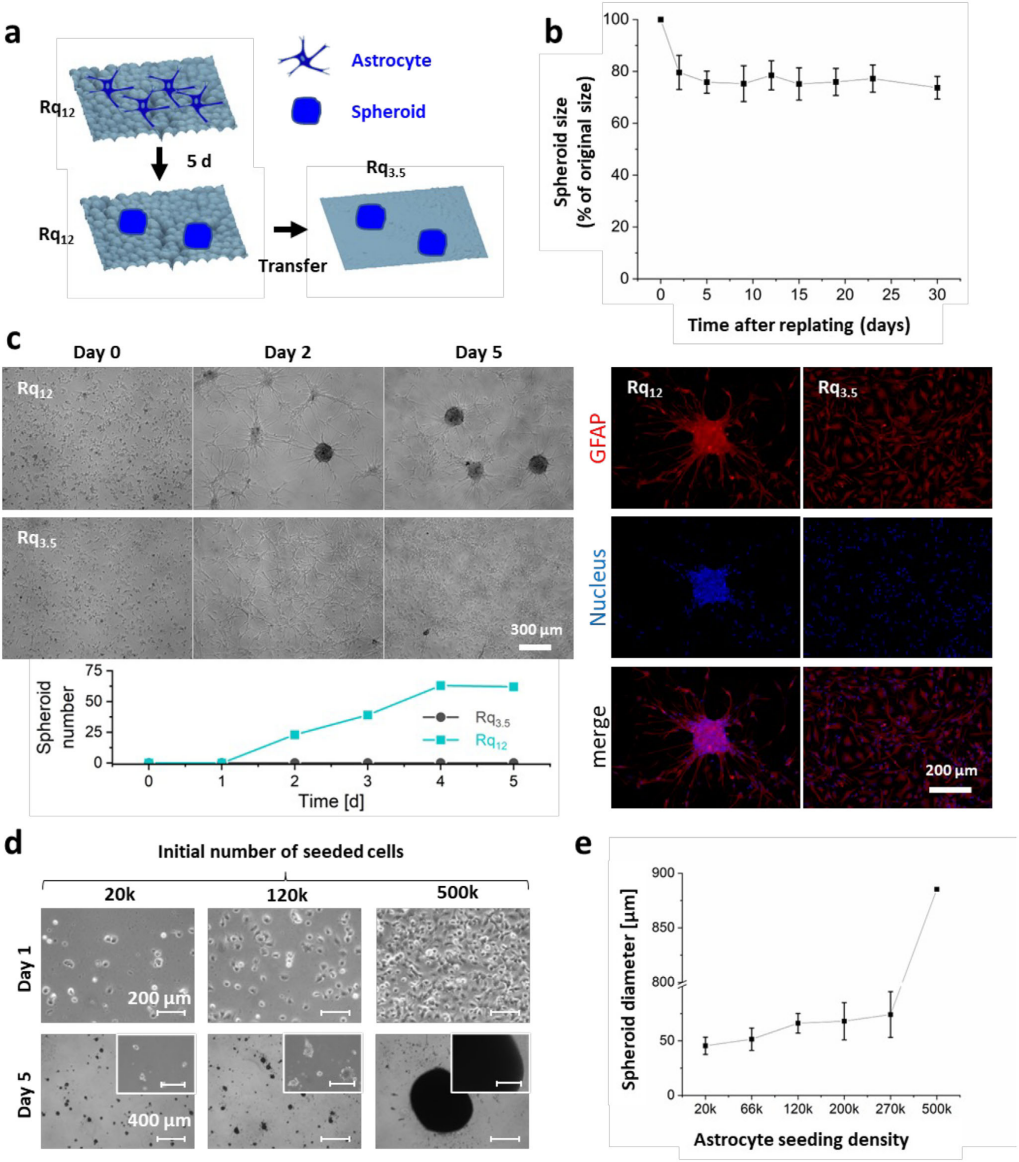
Stochastic nanoroughness‐induced astrocyte spheroids have a stable phenotype. (a) Schematic representation of the replating of astrocytes formed on Rq_12_ onto a smooth Rq_3.5_ substrate in panel b. (b) Size of spheroids formed on Rq_12_ following replating on Rq_3.5_ as a function of days in culture (n = 9). (c) Spheroid formation capacity of astrocytes dissociated from spheroids by collagenase digestion following replating on Rq_12_ and glass substrates (Rq_3.5_), showing that stochastic nanoscale physical cues are essential for spheroid formation. (d) Effect of astrocyte plating density on spheroid size. (e) Quantification of the spheroid diameter as a function of increasing plating density, showing a positive correlation between initial cell numbers and final spheroid size. Error bars represent the standard deviation from the mean (n=9).

Since scar formation by even highly reactive astrocytes, generally assumed to be permanent, is reversible under certain physiological conditions [33, 51], the stability of the astrocyte spheroids induced by nanoroughness was examined. To this aim, astrocyte spheroids matured for 5 days on Rq_12_ were trypsinized without impacting their integrity and transplanted onto glass substrates, which is a smooth surface (Rq 3.5 nm) (Figure 2a). Despite being the trigger for the astrocyte spheroid formation, withdrawal of the Rq_12_ nanoroughness did not lead to spheroid disintegration. Barring a slight reduction from the initial size, which can be attributed to the transplantation process, astonishingly, the spheroids remained intact and stable in their size even after 30 days, implying that the phenotype induced by nanoroughness represents a permanent and fundamental transformation (Figure 2b). To inquire if astrocytes within spheroids retain this phenotype upon dissociation, spheroids were harvested on day 5 from Rq_12_ and dissociated into single cells using collagenase digestion and then replated on both glass (Rq_3.5_) and Rq_12_ substrates. While astrocytes expressed GFAP after dissociation, confirming their astrocytic phenotype, only astrocytes replated on Rq_12_ formed spheroids again (Figure 2c). This provides direct evidence that the nanoscale stochastic physical cues are essential and trigger the formation of spheroids (Figure 2c). The delayed onset (day 2) of spheroid formation on Rq_12_ can be attributed to the recovery of the cells from digestion‐associated trauma.

**FIGURE 3:**
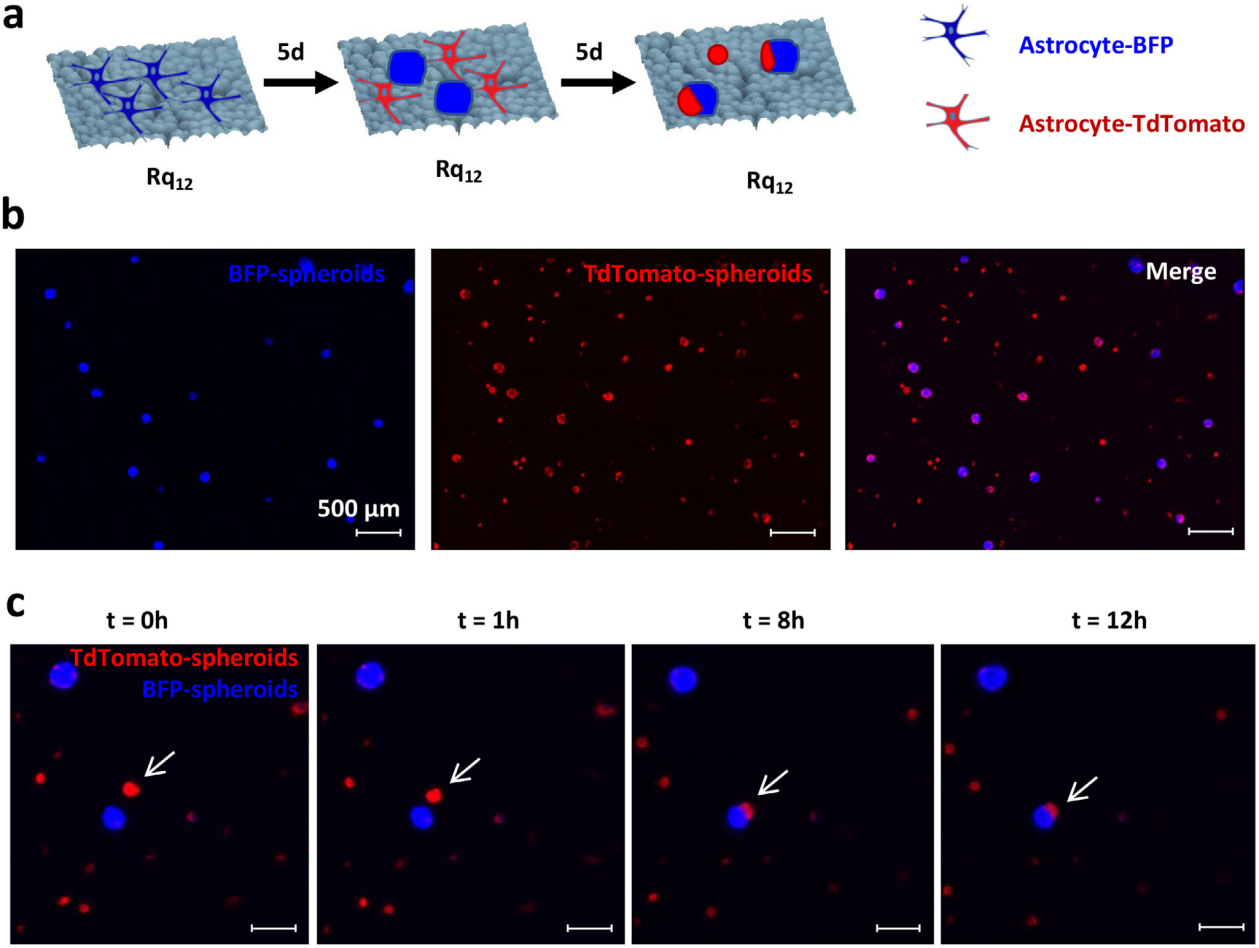
Spheroids function as aggregation centers for naïve astrocytes. (a) Schematic of the experiment design. Substrates were first seeded with BFP‐expressing astrocytes, and after spheroid formation, TdTomato‐expressing astrocytes were introduced to ascertain their interaction. (b) Fluorescence microscopy images taken 5 days after the addition of TdTomato‐expressing astrocytes show 100 % colocalization of BFP fluorescence with TdTomato fluorescence signal, demonstrating the incorporation of TdTomato astrocytes into the pre‐formed BFP‐positive spheroids. (c) Still images from a live‐imaging experiment carried out over 12 h on the fourth day after addition of TdTomato‐expressing astrocytes, capturing a fusion event showing the collective migration of an aggregate of TdTomato astrocytes toward a pre‐existing BFP spheroid, leading to a fusion and incorporation of the TdTomato astrocyte population into the BFP spheroid.

To ascertain the role of cell number, spheroid formation was studied at various plating densities. Increasing astrocyte plating density resulted in a proportional increase in spheroid size with a dramatic (20‐fold) increase above 270k cells/cm^2^ (Figure 2d,e), and at the highest seeding density of 500k cells/cm^2^, the substrate surface was devoid of single astrocytes, and large spheroids were formed (Supplementary Video S1, Supplementary Figure S7). This observation led us to posit that the spheroids are not derived from a single cell but due to the aggregation of distinct astrocytes, which have undergone active association driven by a few astrocytes acting as aggregation centers. To test this hypothesis, an experiment was designed as schematically shown in Figure 3a: astrocytes were first transfected to constitutively express the fluorescent proteins BFP and TdTomato, allowing for simultaneous and independent tracking of both populations. BFP‐expressing astrocytes were seeded on Rq_12_ substrate for 5 days to induce spheroid formation, and then, astrocytes expressing TdTomato were added to the substrates, and the evolution of the spheroids was monitored over 5 days using fluorescence microscopy. BFP‐astrocytes formed spheroids as anticipated, verifying that the transfection did not impede or influence the spheroid formation. Following the addition of TdTomato astrocytes, after 5 days, Td‐astrocytes also formed spheroids by themselves, but many of the Td‐astrocytes fused with the a priori formed BFP‐astrocyte spheroids, with 100% of BFP‐positive spheroids showing the incorporation of TdTomato astrocytes and not vice versa (Figure 3b). In one such fusion event captured in Figure 3c, an aggregate of TdTomato expressing astrocytes can be seen collectively migrating a distance of 50 µm in a matter of 8 h to fuse with the existing BFP expressing spheroid (Supplementary Video S2), suggesting that the colocalization resulted from direct attractive paracrine signals and not random association, and that the established spheroid is stably anchored and serves as foci for the aggregation process.

### 2.3 The Gene Expression Patterns of Astrocytes Grown on Rq12 Emulate Tumoral and Early‐Stage GBM Transcriptional Programs

**FIGURE 4:**
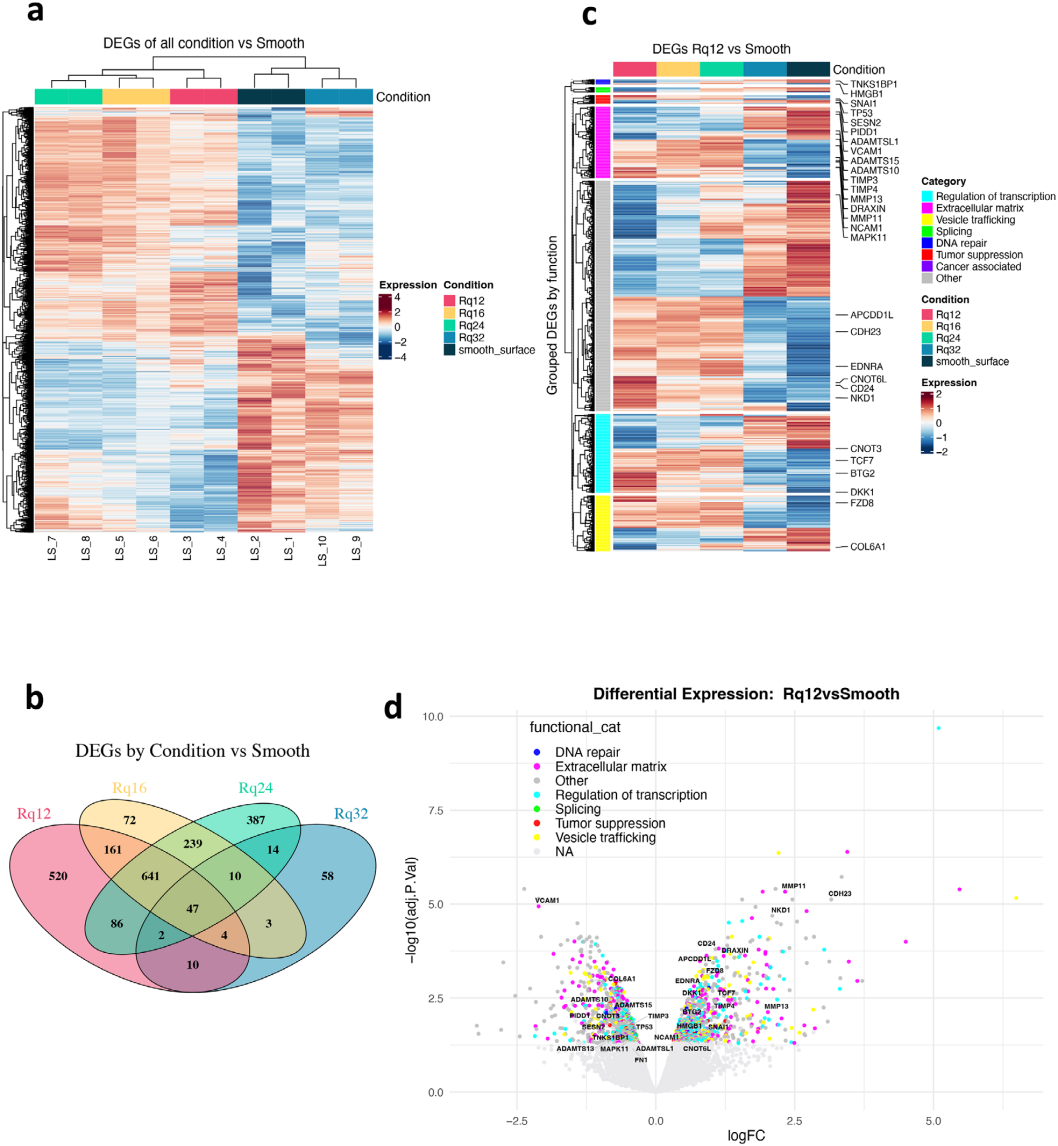
Global transcriptional changes in astrocytes exposed to stochastic nanoroughness. (a) Heatmap of 2254 differentially expressed genes (DEGs) identified in astrocytes in at least one comparison of nanorough substrates (Rq_12_, Rq_16_, Rq_24_, Rq_32_) versus smooth (Rq_3.5_). Values are log2‐counts‐per‐million (log2‐CPM) scaled per gene (Z‐scores). Rows represent genes and columns represent individual samples. The heatmap is clustered hierarchically by both genes and samples; the top bar indicates the condition. Color scale indicates relative expression, from low (blue) to high (red). (b) Venn diagram of DEGs for each nanoroughness condition versus smooth. The diagram shows the overlap of DEGs identified in pairwise comparisons of each nanoroughness condition (Rq_12_, Rq_16_, Rq_24_, Rq_32_) versus the smooth surface. DEGs were defined as protein‐coding genes with an adjusted *p*‐value < 0.05 (FDR (false discovery rate) < 0.05) from the corresponding conditions in a linear model fit. Each ellipse represents the set of DEGs specific to one nanoroughness level. Shared regions (overlaps) indicate genes differentially expressed in multiple conditions. Rq_12_ displays the largest set of unique DEGs (n = 520). (c) Heatmap of DEGs in Rq_12_ compared to smooth surface (adj. *p* < 0.05), displayed across all five conditions. Values are log2‐CPM scaled per gene (Z‐scores). Rows are hierarchically clustered within functional groups and split by the assigned category (left color bar). Representative genes are labeled on the right. (d) Volcano plot of DEGs in Rq_12_ versus smooth, colored by functional category. The plot displays DEGs from the Rq_12_ vs Smooth comparison, with log_2_ fold change on the x‐axis and –log_10_ adjusted *p*‐value on the y‐axis. Each point represents a gene, colored by functional categories. Notable examples of down‐regulated genes include those related to ECM and ECM remodeling (collagens, fibronectin, ADMATS), p53 signaling, and SESN2, which has a known tumor suppressor function. Genes with no assigned category are shown in gray. Label overlaps were limited to improve readability.

Since pathological conditions are often caused by dysfunction of multiple genes and gene networks, a global gene‐expression study using bulk RNA‐seq was performed (workflow shown in Supplementary Figure S8), allowing us to gain unbiased whole‐genome differential gene expression. RNA extracted from astrocytes cultured on Rq_3.5_, Rq_12_, Rq_16_, Rq_24,_ and Rq_32_ for 5 days, that is, the day after marked phenotypical changes are observed and well established, was analyzed. In total, 2254 differentially expressed genes (DEGs) (p < 0.05) were identified in the cells exposed to nanoroughness compared with cells grown on a smooth substrate (Rq_3.5_), which is visualized in the heatmaps and Venn diagram (Figure 4a,b). Among these DEGs, 520 genes (23.07%) were specific to Rq_12_ (Supplementary Figure S9), accounting for the highest proportion among the four stochastic roughness conditions studied. The DEG for Rq_16,_ Rq_24_, and Rq_32,_ and other comparative intersections are shown in Supplementary Figures S10–S15. Unbiased hierarchical clustering grouped samples into two distinct clusters: the two roughness extremes—Rq_3.5_ and Rq_32_ ‐ clustered together and separated from Rq_12_, Rq_16_, and Rq_24_, a pattern further supported by principal component analysis (PCA), where PC1 (40% variance) captured differences driven by stochastic nanoroughness and PC2 (15% variance) reflected minor variation from experimental covariates, indicating minimal hidden effects in the dataset (Supplementary Figure S16). Taken together, these data match the earlier phenotypical observations and emphasize the specificity of the phenotype of astrocytes within the spheroids on Rq_12_. Interestingly, among the 520 DEGs specific to Rq_12_, the majority (64%) were down‐regulated. Based on a study showing that the preponderance of downregulated genes is indicative of dedifferentiation associated with malignant transformation in a cellular model [52], we speculate that astrocytes cultured on Rq_12_ may have undergone dedifferentiation consistent with transformation‐associated cancer‐like phenotype. Functional categorization of DEGs by querying Ensembl via biomaRt to retrieve Gene Ontology (GO) annotations for each Ensembl gene ID [53] enabled the sorting into functional categories (Figure 4c,d). Although it is challenging to determine the influence of individual genes on a biological mechanism, especially when genes within the same category show both down‐ and up‐regulation, general trends could be deduced. A heatmap of DEGs in the Rq_12_ condition versus all other conditions shows downregulation of genes associated with tumor suppression, extracellular matrix (ECM), regulation of transcription, splicing, and vesicle trafficking (Figure 4c). This is further evidenced in the volcano plot of DEGs in Rq_12_ versus smooth condition, which shows downregulation of ECM‐related genes like ADAMTS proteases, collagens, fibronectin‐1, TIMP3, vimentin, and cancer progression‐related proteins such as NOTCH3 and SESN2 (Figure 4d). Moreover, the expression pattern of genes responsible for vesicular trafficking appeared to be biased toward a higher expression of genes regulating extracellular vesicle secretion, one of the known mechanisms by which cancer cells communicate with their surroundings [54]. Several genes involved in alternative splicing were downregulated, while none were upregulated. This is a significant finding as brain tissue is known to exhibit one of the highest degrees of alternatively spliced transcripts, expressing a unique set of isoforms [55]. Furthermore, reprogramming of alternative splicing is a hallmark of cancer, where the tumoral cells express new specific splicing isoforms [56, 57, 58]. Interestingly, 14 genes involved in DNA repair were found to be downregulated, likely leading to genome instability, a characteristic of almost all human cancers [59]. Tumor suppressors overall were downregulated, including novel tumor suppressor candidates found in cervical and gastric cancer, VILL [60], CDK5RAP3 [61], and KIAA0141 [62], along with a decrease in the expression of tumor protein P53 (TP53). TP53 is necessary to maintain a baseline expression of a wide range of tumor suppressor genes [63], and TP53 mutations, leading to loss of p53, were found to be the most frequent and earliest detectable genetic alteration in low‐grade astrocytomas and GBM [64] and contribute to glioblastoma progression [65]. Alterations of p53 were identified in the cancer cells directly but also at the tumor borders in the astrocytes, favoring cancer malignancy [66]. It has been recently shown that the loss of p53 destabilizes astrocyte identity, priming them for dedifferentiation later on in response to inflammatory cues [67]. Furthermore, several other genes implicated in cancer pathology including GBM, namely, TP53‐inducible protein‐11 (TP53I11) [68], MTA1 [69], GLIPR1 [70, 71], GNA13 [72], NCEH1 [73], GMCL1 [74], BEX2 [75, 76], POU2F1, a gene linked to cancer cell survival [77], along with DPP4, which implicated in senescence [78], and LUM, a gene that encodes for the lumican, a PG associated with aggressive GBM, stemness, and ECM remodeling in GBMs [45, 79] were differentially expressed.

**FIGURE 5:**
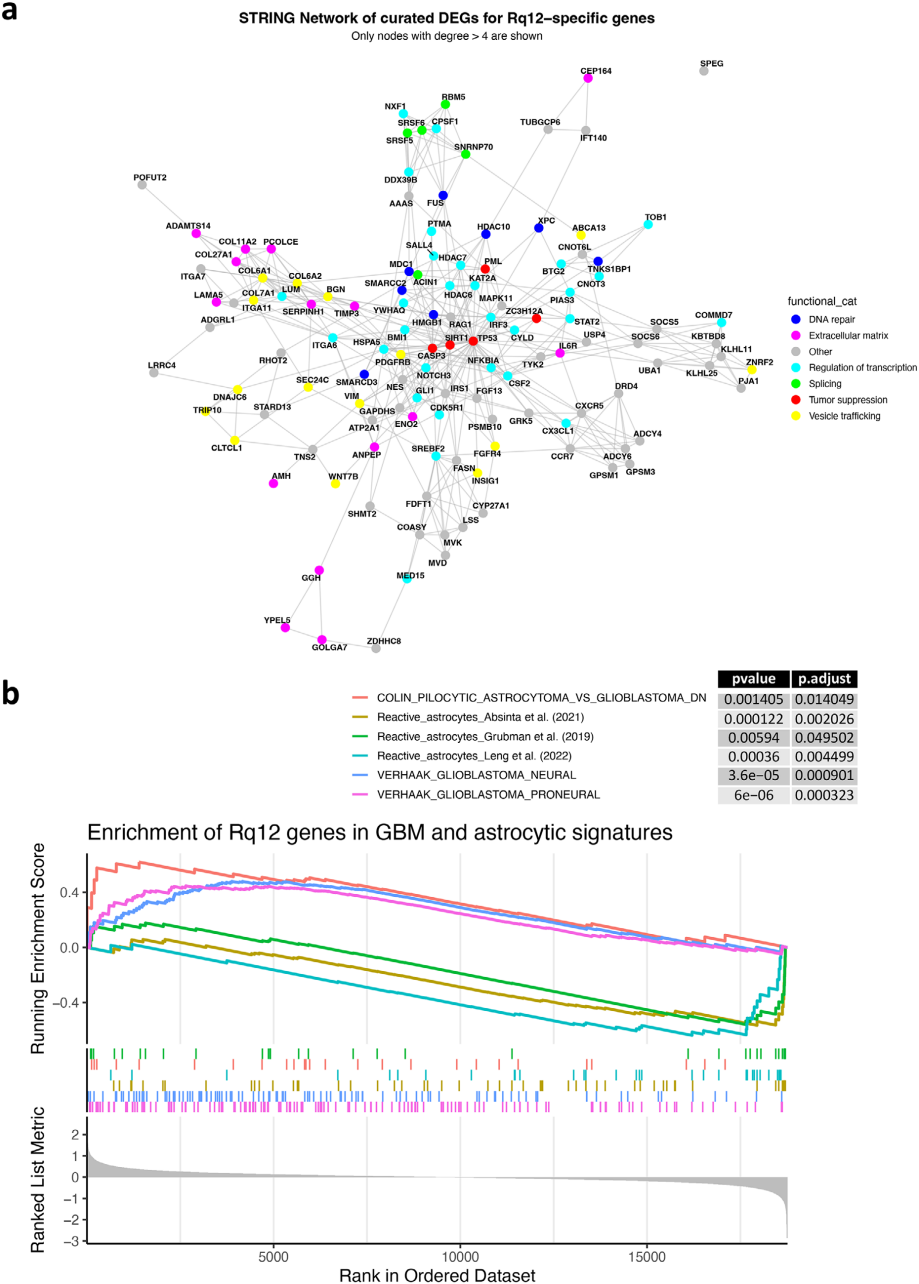
Network and enrichment analyses of Rq_12_ DEGs and analysis of phenotype of astrocytes in nanoroughness‐induced spheroids: (a) STRING Network of Curated DEGs for Rq_12_‐Specific Genes: This network visualizes protein–protein interactions (PPIs) among Rq_12_‐specific DEGs identified from the "Rq_12_ vs Smooth" comparison. Only genes unique to Rq_12_ and showing any expression change (|logFC| > 0) were mapped using the STRING database (score ≥ 400). Nodes represent genes colored by Functional annotations, and edges represent known or predicted PPIs. String analysis reveals nodes involving the TP53 locus, with further involvement of ECM (ADAMTS, COL), and NOTCH3. (b) Gene set enrichment analysis (GSEA) of Rq_12_ astrocytes against GBM, astrocytoma, and reactive astrocyte signatures. Differentially expressed genes from the Rq_12_ vs all other conditions contrast were ranked by log_2_ fold change and tested for enrichment using GSEA against a combined panel of curated signatures, including glioblastoma subtypes, astrocytoma, and reactive astrocyte programs (full list of signatures can be found in Supplementary Figure S17). The running enrichment score (y‐axis) is plotted against the ranked gene list (x‐axis). Only FDR < 0.05 results are shown. The plot shows GSEA enrichment curves for three Verhaak GBM subtype gene sets, with the running enrichment score on the y‐axis and the ranked gene list (ordered by logFC) on the x‐axis. A strong positive enrichment for proneural and neural GBM signatures is observed, as well as an inverse correlation with genes down‐regulated in pilocytic astrocytoma relative to glioblastoma, indicating engagement of glioblastoma‐associated developmental programs. In contrast, several curated reactive astrocyte programs (Absinta 2021, Grubman 2019, Leng 2022) [84, 85, 86] showed significant negative enrichment, demonstrating that Rq_12_ astrocytes diverge from classical injury‐ or inflammation‐induced reactive states.

To fully appreciate how the selected genes work in concert to drive cancer‐related processes, the meta‐database String (version 11.5) was used to map all known evidence‐based interactions between genes to establish a gene interaction network [80], thus allowing for a statistical assessment of the genetic interactions between the DEGs and relationships between the genes. According to the String analysis, with an average node degree of > 4, and an enrichment *p*‐value of 0.00813, the DEGs from Rq_12_ were found to have more interactions among themselves than what would be expected from a random set of proteins of similar size drawn from the genome (Figure 5a). With an increase of the nodes by 20%, such an enrichment indicates that the proteins are at least partially biologically connected as a group. A large node centered around the TP53 was identified. It is worth noting that cancer cells have been shown to transform neighboring astrocytes by reducing their p53 expression, thus shutting down the cell‐death pathways in both cell types, favoring tumor progression [66]. String analysis further revealed three prominent signaling nodes involving ADAMTS proteases, collagens, and NOTCH3, suggesting that these genes may be integral to the phenotype observed in the spheroids. Notch‐signaling, through the Notch intracellular domain (NICD), represents a paracrine, mechanotransduction signaling system that has an important role in cell‐cell adhesion and a demonstrable role in the collective migration of cancer, stromal, and vascular cells in breast cancer models [81]. It has been shown that NICD functions as a cofactor for Lymphoid enhancer‐binding factor 1 (LEF‐1) [82], which is the nuclear target of the canonical Wnt‐pathway, a known regulator of tumorigenesis, with a role in the progression of GBM [83]. The disruption in Notch‐signaling, with the dysregulation of protease networks, can set the stage for tumor‐like traits in the astrocyte spheroids.

To gain further insights into the phenotype of the astrocytes, we performed gene set enrichment analysis (GSEA) using DEGs from the Rq_12_ condition (i.e., within the spheroids) versus all others. We did not use the IVY GBM atlas or TCGA datasets for direct comparison, as both are derived from heterogeneous tumor tissues composed of multiple cell types and lack appropriate non‐tumor astrocyte controls. The IVY atlas, in particular, provides spatially resolved expression within tumors but does not support differential comparisons to healthy tissue. Given these limitations, our approach focuses instead on pathway‐level enrichment to assess whether astrocytes cultured on Rq_12_ engage GBM‐associated and reactive‐astrocyte programs, rather than assigning tumor‐subtype identity. Enrichment was assessed against a broad panel of glioblastoma‐ and astrocyte‐related gene sets curated from the MSigDB C2 and C5 collections and supplemented with external datasets (Supplementary Figure S17). This strategy enabled a context‐appropriate analysis of transformation‐associated trends without implying direct tumor identity. The results demonstrate a significant enrichment of GBM‐associated signature in the Rq_12_ transcriptional profile (Figure 5b). In particular, astrocytes from Rq_12_ spheroids showed strong enrichment for proneural and neural GBM subtype signatures (FDR q < 0.001), whereas mesenchymal and classical signatures were not enriched. The gene sets analyzed and the respective statistical parameters are shown in Supplementary Figure S18. Conversely, several curated reactive astrocyte programs (Absinta 2021; Grubman 2019; Leng 2022) [84, 85, 86] showed significant negative enrichment, indicating that Rq_12_ astrocytes diverge from classical injury‐ or inflammation‐induced reactivity. Importantly, no enrichment was observed for Verhaak Mesenchymal or Classical GBM subtypes, which are associated with proliferative and malignant behavior. Taken together, these findings support the interpretation that Rq_12_ astrocytes adopt a distinct, primed astrocytic state that engages GBM‐associated traits while lacking hallmarks of malignancy. These findings nonetheless support the conclusion that exposure to stochastic nanoroughness (biophysical cues) engages transcriptional programs in astrocytes, consistent with a primed, cancer‐associated state but distinct from overt malignant transformation.

### 2.4 Stochastic Nanoroughness Induces Cellular Senescence and Confers Cancer Stem Cell‐Like Characteristics to Astrocytes

**FIGURE 6:**
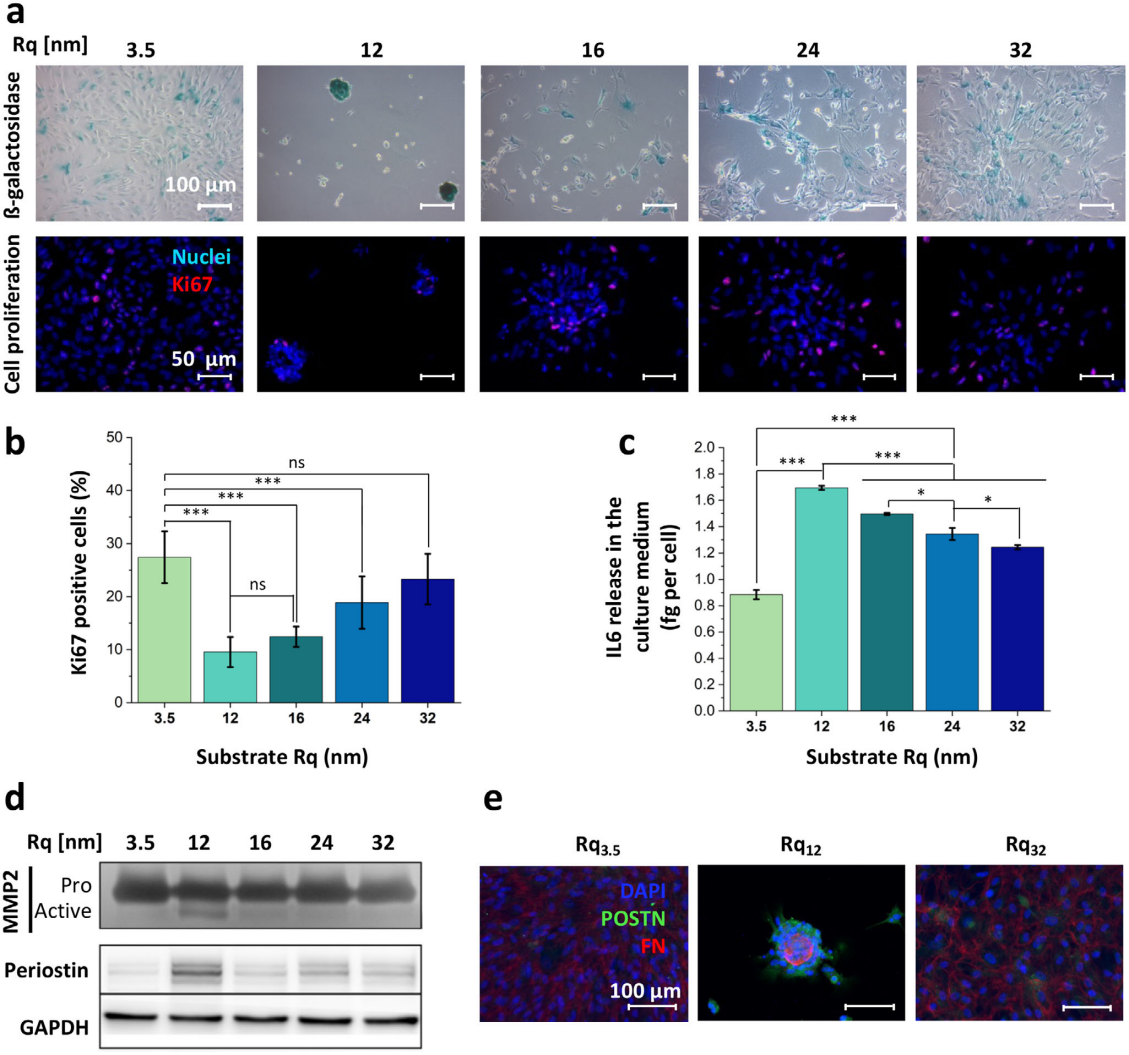
Nanoroughness induces cancer‐associated characteristics in astrocytes. Staining for β‐galactosidase (β‐gal) and Ki67, two “gold standard” markers of cellular senescence revealing a higher β‐gal positivity (a, top panel) and diminished proliferation capacity in astrocytes grown on Rq_12_ (a, bottom panel and b) (Rq_3.5_, n = 8, Rq_12_, n = 11, Rq_16_, n = 4, Rq_24_, n = 9, Rq_32_, n = 7). (c) Quantification of the secretion of IL6, a characteristic and well‐accepted marker for senescence‐associated secretory phenotype in astrocytes, showing enhanced IL6 levels in the milieu of astrocytes grown on Rq_12_ (n = 3). (ANOVA applied with the Tukey test, *p < 0.05, **p < 0.01, ***p < 0.001, ns no statistical difference). (d) MMP2 zymography and western blotting for periostin (POSTN) in astrocytes grown on Rq_12_ showing activation of MMP2 along with an increase in periostin expression. GAPDH was used as an internal loading control (pooled extracts from n=3). The entire gel and western blot for the proteins are shown in Supplementary Figure S17. (e) Immunofluorescence staining for persiotin in astrocytes grown on Rq_12_ reveals a higher expression in this population in comparison to astrocytes grown on Rq_3.5_.

Senescence, the arrest of the cell cycle, wherein cells are incapable of cell division but are metabolically active, is crucial during embryonic development, and tissue remodeling, including wound‐healing, has been implicated in cancer, and it plays a role in preventing the proliferation of cells experiencing oncogenic stress [87]. Since astrocyte spheroids exhibit many traits of GMB and related transcriptional programs, we inquired whether stochastic nanoroughness could trigger senescence. In contrast to those entering apoptosis, cells entering senescence remain viable and exhibit a senescence‐associated secretory phenotype (SASP) [87]. The “gold standard” biomarker for identifying senescence is the senescence‐associated beta‐galactosidase reactivity (SA‐β‐gal) [87, 88, 89], also expressed by aging neuronal cells [90]. Astrocytes cultured on the different roughness regimes were stained for β‐gal, and spheroids formed on Rq_12_ were strongly positive for β‐gal, suggesting that they have undergone senescence. The lower percentage of Ki67‐positive cells provided further strong evidence that the astrocytes had undergone senescence (Figure 6a,b) [87]. The induction of senescence is consistent with the observation that spheroid size remained constant after replating Figure 2b. Since senescent cells are known to display a unique SASP phenotype, which has potentially detrimental long‐term implications [91], the release of the cytokine IL6 was investigated. Senescence‐associated IL‐6 expression has been implicated in the induction of an inflammatory environment and tumorigenic traits in MCF‐7 breast cancer cells [91]. While IL6 expression was observed in all nanoroughness conditions, a significantly higher IL6 production per cell was found in the Rq_12_ condition, characteristic of SASP (Figure 6c). This is a significant finding as it is the first report of astrocyte senescence triggered by physical cues in contrast to environmental triggers such as oxidative stress, radiation, hyperoxia, oncogenes (RAS or Raf), and replicative exhaustion [92, 93, 94, 95]. Interestingly, elevated levels of IL6 have been observed around closed‐head injuries [96], and also detected in cerebrospinal fluids [96, 97].

Matrix metalloproteinases (MMPs) expression is dysregulated in several pathological conditions, particularly cancer, where they have been one of the most researched therapeutic targets and biomarkers [98]. Since senescent cancer‐associated fibroblasts have been shown to secrete pro‐MMP‐2 [99], and in the CNS, MMPs are mainly produced by astrocytes, particularly in response to injury‐related activation [100], the influence of nanoroughness on astrocytic release of MMP‐2 was investigated. Although the roughness regimen did not influence pro‐MMP2 release, active‐MMP‐2 was uniquely detected in the culture medium of astrocytes grown only on Rq_12_ (Figure 6d, Supplementary Figure S19). A positive correlation between astrocyte seeding density and the amount of activated MMP‐2 evidenced that astrocytes are directly involved in the activation process (Supplementary Figures S20 and S21), demonstrating MMP‐2 activation as a consequence of astrocyte senescence. This is the first evidence of MMP‐2 activation by astrocytes directly triggered by physical cues, which might involve astrocyte senescence, and provides further evidence for brain ECM topography as one of the contributing factors in neurodegeneration [32]. MMP‐2 can localize on the surface of invasive cells via integrin binding and promote cell‐mediated collagen degradation [101], and reactive astrocytes have been detected around gliomas [15, 18]. Also, activation of MMP‐2 secreted by astrocytes requires interaction with glioma cells [102]. Based on these observations, we suggest that the ability of senescent cells to shape the microenvironment is not limited to cytokine and protease secretion but may also include differential ECM production. We therefore investigated the secretion of periostin (POSTN), a matricellular ECM protein with a known role in tissue remodeling [103], and cancer progression and metastasis [104, 105], whose expression in cancers is associated with poor prognosis. A strong upregulation of POSTN by astrocytes cultured on Rq_12_ (Figure 6d) was verified by Western blot analysis and by immunohistochemistry (Figure 6e), with spheroids induced by Rq_12_ showing prominent and uniform staining for POSTN throughout the entire spheroid (Supplementary Figure S22). Additionally, a staining for fibronectin was observed in the periphery of the spheroid. It has been shown that fibronectin expression positively correlates with glioblastoma aggressiveness by promoting cell cohesion and collective invasion in orthotopic tumors in mice [106]. However, astrocytes cultured on Rq_3.5_ and Rq_32_ showed no such expression, providing further evidence that astrocytes in the spheroid represent a unique phenotype. Since high POSTN expression has been observed in tissues (periosteum, periodontal ligament) subjected to constant mechanical stress, it is thought to play a role in mechanoreception, and its upregulation in astrocytes exposed to nanoroughness reinforces this postulated role [107, 108]. It has been shown that stochastic nanoroughness alters the membrane distribution of the stretch‐activated, mechanosensing ion channel Piezo‐1 in neurons [32]. Furthermore, as POSTN expression is low under healthy conditions but upregulated following injuries [109], the prominent expression of POSTN by astrocytes grown on Rq_12_ represents a hitherto unexplored marker for activated astrocytes.

Reactome pathway enrichment analysis of upregulated genes (logFC > 0.5) in Rq_12_ astrocytes, compared to the other substrates, showed prominent enrichment in ECM‐related pathways (organization, degradation, proteoglycans) and inflammatory lipid mediators (EET, HETE), supporting the observed phenotypic shift toward a tumor‐like and SASP state (Supplementary Figure S23). These molecular signatures align with morphological and functional traits of GBM‐associated astrocytes described in this study. Furthermore, reactome pathway enrichment analysis of significantly downregulated genes (logFC < –0.5) in Rq_12_ astrocytes relative to all other substrates showed marked suppression of collagen‐related biosynthesis, ECM assembly, mitochondrial respiration, and key signaling processes (e.g., GPCR, PDGF). This pattern suggests a dedifferentiated, ECM‐disengaged, and metabolically reprogrammed phenotype consistent with transformation toward a tumor‐ or senescence‐associated astrocyte state.

### 2.5 Stochastic Nanoroughness Mediates the Crosstalk Between Astrocytes and Cancer Cells Through the Induced Cell Senescence

**FIGURE 7:**
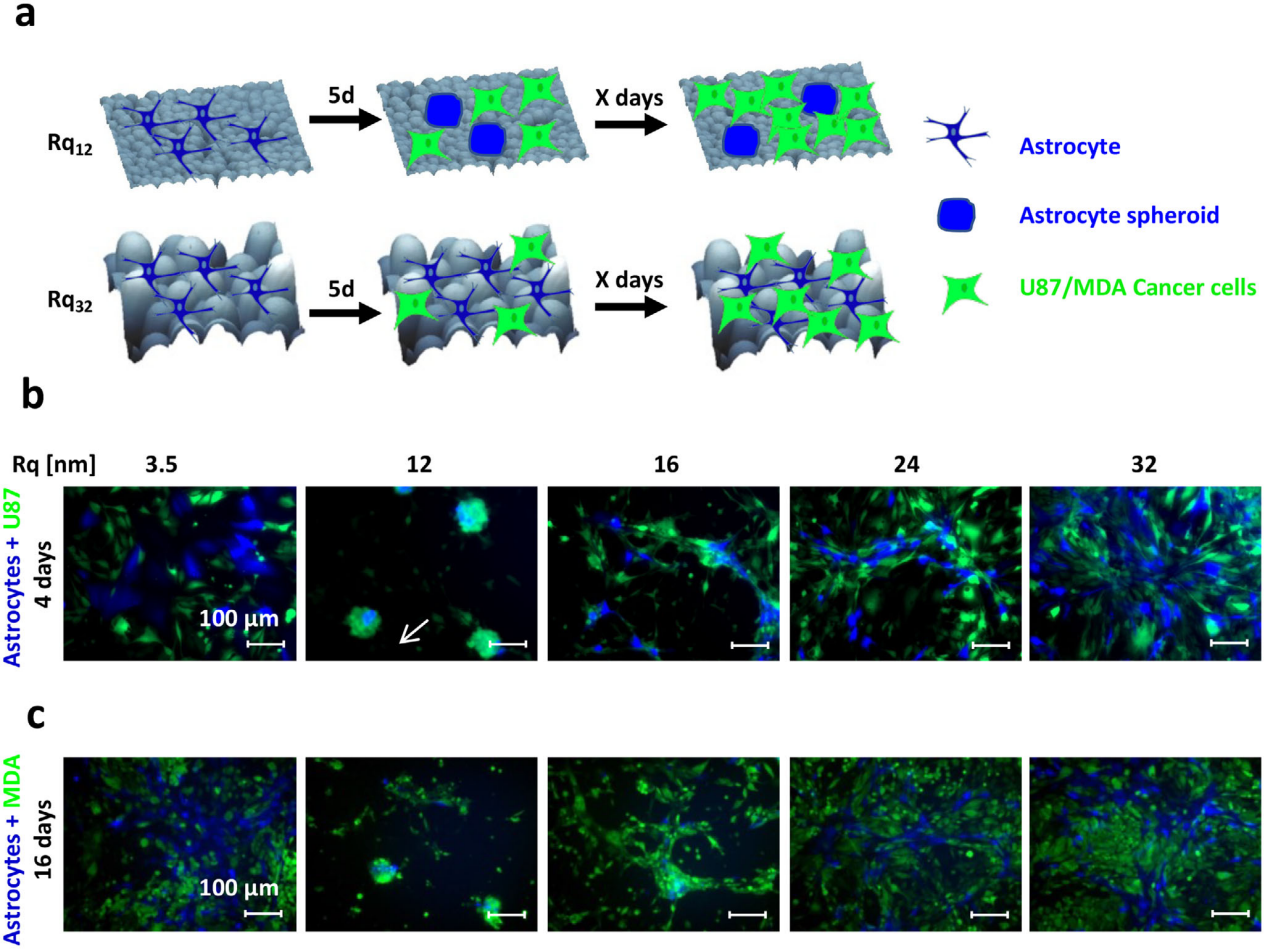
Stochastic nanoroughness mediates the interactions between astrocytes and cancer cells. (a) Schematic representation of the experimental design. Naïve astrocytes were cultured for 5 days to establish the respective phenotype on varying stochastic nanoroughness, and then cancer cells, U87 or MDA‐MB‐231, were seeded, and their fate was followed until clear trends emerged. (b) Fluorescence microscopy images after 4 days of co‐culture of BFP expressing astrocytes and green fluorescent protein (GFP) expressing U87 cells and, (c) Fluorescence microscopy images after 16 days of co‐culture of BFP expressing astrocytes and GFP expressing MDA‐MBA‐231 cells, showing associative tendencies between astrocyte spheroids on Rq_12_ substrates and U87 and MDA cells, further confirming the unique microenvironment of the astrocyte spheroids formed on Rq_12_.

Senescent tumor cells have been shown to lead collective invasion in thyroid cancer [110], and MMPs secreted by senescent fibroblasts were found to support tumor xenograft growth [111]. Furthermore, senescent cells act as aggregation centers for cancer cells, therefore participating in 3D cluster formation [112] through a mechanism involving the SASP. Building on these findings and our observation that spheroids serve as aggregation centers for naïve astrocytes, we posit that astrocyte senescence, mediated by nanoroughness, may provide attractive cues to invading or metastatic tumor cells. To test this, co‐cultures of astrocytes with U87, a GBM cell line, and MDA‐MB‐231, a triple‐negative breast cancer cell line, were studied on surfaces of varying nanoroughness (Figure 7a). After 4 days of co‐culture, U87 cells were homogenously distributed on Rq's except Rq_12_, where they aggregated around the astrocyte spheroids (Figure 7b). Similarly, after 16 days, MDA‐MB‐231 cells were also associated with the astrocyte spheroids when co‐cultured on Rq_12_ (Figure 7c), suggesting that the cancer cells are drawn toward the spheroid microenvironment in both systems. Since both U87 and MDA‐MB‐231 cells, when cultured on their own over the same duration, did not exhibit discernible changes in phenotypical traits due to nanoroughness (Supplementary Figure S24), the associative tendency between the cancer cells and astrocyte spheroids on Rq_12_ can be attributed to the astrocyte phenotype. Because breast cancer, together with melanoma and lung cancer, are the three tumor types known to metastasize to the brain [113], we conclude here that astrocytes and nanoroughness may synergistically function as a specialized niche microenvironment to attract circulating tumor cells and promote metastatic lesions [114]. Our results also reinforce findings from a previous study showing the ability of astrocytes to alter the migration and morphology of metastatic breast cancer cells [115]. To rule out that cancer cells are guided toward the astrocyte spheroids by ECM tracks, the opposite experiment was performed, where astrocyte spheroids formed on Rq_12_ were trypsinized, and the intact spheroids were transferred onto U87 cells cultured on smooth Rq_3.5._ Here too, U87 cells migrated toward the astrocyte spheroids after 48 h of co‐culture, confirming the attraction of cancer cells toward the senescent astrocyte population in the spheroids (Supplementary Figure S25a,b). This experiment, performed on a smooth (Rq_3.5_) substrate, confirmed that the effect of nanoroughness on the crosstalk is rather indirect, through the durable transformation of astrocyte phenotype to SASP. The attraction of U87 cells to the astrocyte spheroids was however not accompanied by an increase in proliferation as assessed by the MTS assay (Supplementary Figure S25c), suggesting that interactions between U87 and astrocytes are attractive, and do not involve increased local proliferation, further supporting that the SASP astrocyte phenotype exhibits unique crosstalk with cancer cells. These findings are highly significant; they present evidence for a biophysical basis for the induction of oncogenic transcriptional changes in astrocytes and provide the impetus to investigate the role of astrocyte aggregation in the induction of an aberrant phenotype in the astroglial population. In a preliminary experiment, we have shown that astrocytes dissociated from spheroids grown on Rq_12_ after stereotaxic implantation in mice brains remarkably survive for over 12 months and disseminate (migrate) away from the site of injection (Supplementary Figure S26) as assessed by staining for Alu‐repeat, which is specific for human nuclei. The absence of tumor induction in mice is consistent with our observations that astrocytes dissociated from spheroids do not reform spheroids in the absence of a biophysical trigger, which we theorize could evolve after a TBI. Future experiments will explore the direct injection of intact spheroids and the co‐injection of astrocytes grown on the different nanoroughness with GBM cells to assess whether the astrocytes grown on Rq_12_ can facilitate tumor progression in vivo.

## 3 Discussion

As opposed to deterministic topography (gratings, lines, pillars), which are periodic and well‐defined and do not occur in mammalian physiology, stochastic topography is a hallmark of the physical environment defined by ECM and thus is physiologically relevant. Therefore, our findings that stochastic nanoroughness (nanotopography) can induce the spontaneous association of naïve astrocyte into spheroids, and induce transcription traits observed in proneural GBM are quite significant as they provide evidence for a potential biophysical basis for the formation of glioma; and a plausible mechanism to underpin the cause‐and‐effect link between TBI and GBM. While the molecular mechanism of spheroid formation remains to be unraveled, factors that promote cell‐cell interaction over cell‐substrate interactions could be one factor, as observed in GMB cell lines [116]. The ability of spheroid‐derived astrocytes to survive implantation and migrate in the mouse brain (Supplementary Figure S26) implies that astrocytes exposed to physical cues can persist in vivo and engage distant locations. When placed in the context of our earlier findings that the topography of the brain tissue is altered around Alzheimer's plaques [32], and that PGs contribute prominently to the mechanical properties of the brain [37], the evidence presented here provides a compelling argument to consider changes to the microstructure of the brain in the induction of neurodegenerative conditions. To elucidate the underlying molecular mechanisms that might lead to astrocyte aggregation, further studies could examine outcomes between surfaces that present deterministic roughness (pillars, lines) in the same length scales as the R_q_ values investigated in this study. Since inflammatory processes and biochemical changes associated with TBI are not captured by the stochastic nanoroughness model, future studies should include temporal presentation of soluble cues using delivery systems [117]. Also, a time course analysis of changes to astrocyte phenotype within spheroids using single cell omics would further shed light on the extent to which the astrocytes adopt GBM‐like transcription traits and when a GBM‐like phenotype might emerge. Additionally, to gain further insights into the phenotype of astrocytes upon exposure to stochastic biophysical cues, comparative transcriptomics using GBM samples from patients would be a necessary next step to shed light on the significance of the findings for developing in vitro models.

Among the many pro‐oncogenic factors, NOTCH (1–4) signaling plays a critical role in tumorigenesis [118, 119] and the progression and invasion of GBM [120]. NOTCH signaling is a highly conserved juxtacrine signaling paradigm that requires cell‐cell contact [121]. NOTCH3 has been shown to correlate with poor prognosis and increased invasive traits in GBM [122]. It has been shown that breast cancer cell phenotype mediates mesenchymal‐NOTCH signaling via mesenchymal‐endothelial crosstalk [81]. The downregulation of NOTCH3 in the astrocyte spheroids suggests that gaining invasive traits might require the acquisition of vasculature and immune cells to fulfill juxtacrine signaling. In addition to soluble cues, extracellular matrix FN expression has been associated with the progression [123] and invasion of gliomas [106], and gene ontology enrichment analysis has revealed that FN and ECM‐related genes are upregulated in GBM tissue versus healthy brains [124]. Here, a downregulation in FN expression was observed in astrocytes within spheroids, suggesting that other extraneous factors, only accessible in the in vivo environment, are necessary to drive an aggressive malignant phenotype. The gene interaction analysis reveals an astrocyte phenotype within the spheroids with many cancer‐related traits. In future studies, we aim to elucidate the role of microglia and crosstalk between endothelial cells derived from glioma environments and activated monocytes in driving the maturation of the astrocyte spheroids into a malignant phenotype, with pronounced GBM‐like invasive traits.

Considering the findings of this study, the idea of conditioned astrocytes (defined as a reactive astrocyte population) as a therapeutic tool in treating neurodegenerative conditions is worth considering. Toward this objective, a detailed analysis of the epigenome of astrocytes exposed or conditioned to biophysical cues could identify new targets for mitigating malevolent changes to astrocyte physiology. Furthermore, one could also envision the implantation of astrocytes conditioned ex vivo using physical cues to predictably alter cellular and signaling homeostasis and bestow protective traits in the brain.

In conclusion, by elucidating the role of physical cues, our study provides novel insights into how the microstructure of the brain may play a pivotal role in modulating astrocyte behavior and contributes to a better understanding of the complex interplay between biophysical factors and soluble signals in tumor induction in the brain.

## 4 Materials and Methods

### 4.1 Cells

Human fetal astrocytes isolated from cerebral cortex (passage 1) were procured from Sciencell (product #1800) and tested by the supplier and confirmed to be negative for HIV‐1, HBV, HCV, mycoplasma, bacteria, yeast, and fungi. Astrocytes were cultured in complete astrocyte medium (Sciencell, including 2% FBS, catalog #1801) in a humidified incubator at 37°C and 5% CO_2_. For expansion, cell adherence was ensured by coating the tissue culture plastic plate with Poly‐L‐Lysine at 2 µg/cm^2^. Human glioblastoma‐derived cell line, U87, was purchased from ATCC and cultured in DMEM + 10% FBS in a humidified incubator at 37°C and 5% CO_2_. The human triple‐negative breast cancer cell line MDA‐MB‐231 was purchased from ATCC and cultured in DMEM + 10% FBS in a humidified incubator at 37°C and 5% CO_2_. All cell lines were genotyped and tested negative for Mycoplasma. Studies were replicated using two independent astrocyte donors. All experiments were performed using astrocytes at passage three.

### 4.2 Transfection

Lentiviral particles containing BFP: pLVX‐mTagBFP2‐P2A‐Puro (BIOSS Toolbox, University of Freiburg), GFP: pGIPZ (Openbiosystems, RHS4 346), and tdTomato (pLVX‐tdTomato, BIOSS Toolbox, University of Freiburg) were produced in HEK293 cells by mixing lentiviral vector and packaging vectors using branched polyethyleneimine (bPEI) (MW 25 KDa, Sigma) as the transfection reagent. For transfection, 5 µg of DNA (4:3:1 of a transgene, pCMVdR8.74 (packaging plasmid; Addgene, Plasmid #22036) and pMD2.G (envelope plasmid, Addgene, Plasmid #12259) were diluted in 250 µL Opti‐MEM (Invitrogen), afterward, 11.25 µL of bPEI (1 mg/mL) was added and incubated for 25 min at room temperature before transferring to HEK293 cells. 16 h after the transfection, the medium was exchanged with the medium of the target cells, and 24 and 48 h after that, the media containing lentiviral particles were collected and filtered through a sterile 0.20 µm syringe filter (Millipore) to infect the target cells. Infected cells include the human fetal astrocytes, U87, and MDA‐MB‐231 cell lines. 3 days after transduction, infected cells were selected by adding puromycin (2 µg/mL) (Sigma) to the culture medium.

### 4.3 Preparation of Surfaces Presenting Stochastic Roughness

Surfaces presenting stochastic nanoroughness were prepared by spin‐coating silica (SiO_2_) nanoparticles (SiNP) on glass substrates (Supplementary Figure S1). Briefly, SiNPs were synthesized using the Stöber process as described earlier [30, 32, 125] via ammonia‐catalyzed hydrolysis of tetraethylorthosilicate (TEOS) in 200‐proof ethanol. SiNP size was tuned by varying the amount of ammonia and TEOS in the reaction. The size of the SiNP was confirmed using dynamic light scattering (DLS) analysis performed on DelsaNano C equipped with the DelsaNano Software V3.73/2.3 (Beckman Coulter Inc.) in triplicate. Then, glass slides of 5cm^2^ (2.5 × 2.0 cm) were cleaned by successive immersion in toluene—acetone—ethanol for 5 min each in a slide holder placed an ultrasonic bath, and following a drying period of 30 min at 60°C, 200 µL of SiNP solution of the desired size was deposited at low‐speed (5s, 400 RPM) followed by film casting (20s, 2000 RPM) using a spin coating (P6700 Series; Specialty Coating Systems, Inc.; Indianapolis, USA). To ensure homogeneous coatings, seven layers were deposited, and then the substrates were dried for 12 h at 200°C. The presence of nanoroughness was confirmed using tapping mode on Dimension V Bruker AFM equipped with phosphorus silica doped cantilever (40N/m, 300 kHz, Symmetric Tip), at a scan rate of 0.9 Hz with 256 scans per image. The root‐mean‐square of the roughness (R_q_) was measured and is reported as an average of measurements at five different positions over an area of 1 µm^2^ at five different positions and calculated using NanoScope Analysis 1.20.

### 4.4 Seeding of Astrocytes on Nanorough Substrates

Substrates were placed in 6‐well plates or 35 mm tissue culture dishes and sterilized for 1 h under UV light. Coating with poly‐ornithine (20 µg/mL; 4 µg/cm^2^) and/or laminin (5 µg/mL; 1 µg/cm^2^) was accomplished by incubating for 1 and 2 h, respectively, at 37°C. Geltrex coating was performed according to the manufacturer's protocol (Thermo Fisher Scientific, USA). Astrocyte suspension (66 000 cells/100 µL) was incubated on each substrate for 30 min to ensure optimal attachment, and then 2 mL of astrocyte medium was added (Supplementary Figure S1).

### 4.5 Microscopy

Immunofluorescence staining: Cells were fixed in 3.7% formaldehyde for 15 min, and then blocked with 2.5% goat serum, 0.1% Triton‐X, and 0.05% Tween 20 in PBS for 1 h at room temperature. Four markers were stained using the following primary antibodies: Ki67 (Abcam, 1:500 dilution), POSTN (Abcam, 1:200 dilution), Fibronectin (Santa Cruz Biotechnology, 1:200 dilution), GFAP (Novus Biologicals, 1:1000). The primary antibody was incubated overnight at 4°C, then a secondary Alexa‐conjugated or Cy3‐conjugated antibody was incubated for 75 min at room temperature. Cell nuclei were stained using 1:5000 Hoechst (Invitrogen) solution in PBS for 15 min at room temperature before samples were mounted in Vectashield Vibrance Antifade (Vector Labs). Alternatively, VECTASHIELD DAPI mounting medium (Vector Laboratories, CA, USA) was used to stain the nuclei. Actin staining: For visualization of the actin cytoskeleton, astrocytes were fixed in 3.7% formaldehyde for 15 min, permeabilized in 0.1% Triton‐X in PBS for 15 min, and incubated in 165 nM solution of Phalloidin conjugated with fluorochrome Alexa‐488 (Invitrogen) for 30 min. Cell nuclei were stained using 1:5000 Hoechst (Invitrogen) solution in PBS for 15 min at room temperature before samples were mounted in Vectashield Vibrance Antifade (Vector Labs).

### 4.6 Fluorescence Microscopy

Cells were visualized using a Zeiss Axio Observer Z1 (Carl Zeiss, Oberkochen, Germany) fluorescence microscope equipped with a z‐stage, COLIBRI LED fluorescence high‐speed imaging system, and an on‐stage incubator. Images were acquired and processed using Zeiss Zen Blue (v2.6) software provided by the manufacturer.

Scanning electron microscopy: Scanning electron microscopy images of cells on substrates were obtained as follows. Cells were fixed in 2.5% glutaraldehyde for 30 min, washed in PBS, and dehydrated by incubation for 3 min each in increasing ethanol concentration (10%, 20%, 40%, 60%, 80%, 100%). After an overnight drying, fixed cells on their glass substrate were coated with a gold layer before imaging in a Quanta 250 FEG SEM equipped with the FEI x T software.

Live‐Dead staining: For live/dead staining (LIVE/DEAD Viability/Cytotoxicity Kit, Invitrogen, Waltham, MA, USA), cells on TCP or Rq_3.5_ or spheroids were gently washed once with DPBS and stained with 2 µM Calcein AM (live) and 5 µM ethidium homodimer‐1 (EthD‐1, dead) in DPBS solution for 30–60 min at 37°C and 5% CO_2_.

Spheroid viability: The viability of spheroids was calculated over the volume of cells in a spheroid from images acquired on a Zeiss Axio Observer Z1 (Carl Zeiss, Oberkochen, Germany) fluorescence microscope equipped with a z‐stage and an on‐stage incubator. The images were acquired and processed using Zeiss Zen Blue (v2.6) software provided by the manufacturer. Individual cell sizes were measured, and live/dead cells were counted. Cells were assumed to be spherical. The total volume of spheroids was measured with the assumption of an ellipsoid shape and was set concerning live/dead cells for viability assessment.

Spheroid imaging: Entire spheroids were incubated in 2 µM Calcein AM and 5 µM ethidium homodimer‐1 solution for 30 min and then immediately imaged using a confocal ZEISS LSM 880 Observer equipped with the software ZEN Black 2.3 at the University of Freiburg Live Imaging Center.

β‐Galactosidase staining: Staining for β‐galactosidase was carried out using a commercial kit (Cell Signaling Technology) following the manufacturer's instructions. Astrocytes were fixed for 15 min in the supplied fixing solution, then incubated with the staining solution adjusted to pH = 6 overnight at 37°C, before imaging.

ELISA for IL6: Conditioned media were collected after 5 days of culture and centrifuged at 600 g for 10 min to remove cell debris. The concentration of IL6 was determined using a commercial ELISA kit according to the manufacturer's instructions (R&D Systems, #DY206).

### 4.7 RNA‐Sequencing and RNA‐Seq‐Analysis

The source RNA for the sequencing runs was extracted using the RNeasy Micro kit (Qiagen), and nucleic acid concentration was measured using a Nanodrop 2000c. RNA was used to prepare poly‐A selected directional libraries using the NEB Next Ultra Directional RNA Library Prep Kit (New England BioLabs, USA), and libraries were sequenced utilizing the Illumina HiSeq2000 platform at the sequencing core facility of the Max Planck Institute of Immunology and Epigenetics (Freiburg, Germany). Paired‐end reads were generated from sequencing on the Illumina HiSeq2000. An Overview of the RNA‐Seq computational analysis pipeline can be found in Supplementary Figure S8.

RNA‐Seq analysis: Raw RNA‐seq data (75 bp paired‐end reads) were processed on the sciCORE HPC cluster using a Snakemake‐based workflow (v6 or higher) with conda‐managed environments. Reads were aligned to the human reference genome (hg38) using STAR (v2.6.0b‐1) with settings optimized for reproducible alignment of multi‐mapping reads. Genome indices were built from a UCSC‐derived FASTA file filtered to exclude haplotype sequences, and a GTF annotation based on Ensembl release 112 converted to UCSC coordinates. Aligned reads were sorted by coordinate, merged per sample, and PCR duplicates were marked. Gene‐level quantification was performed using featureCounts (v2.0.0) in paired‐end, unstranded mode. Quantification of spliced and unspliced transcripts was enabled using an intron‐aware model with a read length of 75 bp. Gene body coverage was computed using 100 bins per gene. All configuration and sample metadata were managed via structured YAML and TSV files to ensure full reproducibility.

Gene‐level count data were imported into R and processed using the edgeR (v3.40.0) and limma (v3.54.0) Bioconductor packages. Genes with low expression were filtered using filterByExpr() based on group structure to retain only those expressed above background in at least some experimental conditions. Library sizes were normalized using the trimmed mean of M‐values (TMM) method. Principal component analysis (PCA) was used to assess sample relationships and variance structure. Generalized linear models were fitted with glmQLFit(), and quasi‐likelihood dispersions were visualized to assess model fit. For differential expression analysis, log2‐transformed counts per million (CPM) were computed, and linear models were fitted using lmFit() followed by empirical Bayes moderation with eBayes() using robust = TRUE. Multiple contrasts were defined to compare nanoroughness conditions (Rq_12_, Rq_16_, Rq_24_, Rq_32_) to each other and the smooth surface reference. Differentially expressed genes were identified based on moderated t‐statistics and false discovery rate (FDR)‐adjusted *p*‐values. Gene‐level results, including coefficients and test statistics, were saved for downstream enrichment and visualization.

DEGs were functionally annotated using biomaRt (Ensembl, hsapiens_gene_ensembl). For each Ensembl gene ID we retrieved hgnc_symbol, go_id, name_1006 (GO term name), and namespace_1003 (BP/MF/CC). GO terms were collapsed into broad functional classes relevant to our study (DNA repair, extracellular matrix, regulation of transcription, splicing, vesicle trafficking), and these class labels were used to group/color genes in the heatmap, volcano plots and STRING network.

STRING network analysis on protein‐coding genes uniquely differentially expressed in Rq_12_ compared to all other conditions (Rq_16_, Rq_24_, Rq_32_, and smooth surface) was performed to investigate protein‐protein interaction (PPI) networks associated with condition Rq_12_. Genes were then mapped to STRING identifiers using the STRINGdb R package (v11, human species, score threshold = 400), and unmapped genes were discarded. Protein‐protein interaction data for the mapped genes were retrieved from the STRING database. Redundant or self‐loop edges were removed, and the resulting interaction list was used to build an undirected graph using the igraph package. The network was visualized using the ggraph and tidygraph packages. To improve interpretability, only nodes with degree > 4 were retained in the final graph. Nodes were colored according to their assigned functional category, and gene symbols were displayed as labels. The network layout was computed using the Fruchterman‐Reingold algorithm.

Transcriptional analysis: To assess transcriptional resemblance to GBM, we performed gene set enrichment analysis (GSEA) using protein‐coding DEGs ranked by logFC from the Rq_12_ vs ALL comparison. Gene sets were selected from the MSigDB (v2024.1) C2 and C5 collections, filtering for terms containing “GBM,” “glioblastoma,” “astrocytoma,” or “astrocyte” in their names or descriptions. Sets unrelated to biological context (e.g., drug‐treated non‐neural cell lines) were excluded. To broaden the analysis and minimize bias, we additionally incorporated curated reactive astrocyte programs from recent single‐cell studies by Matusova et al., [126]. In total, 61 signatures (Supplementary Figure S17) spanning GBM subtypes, astrocytoma/low‐grade glioma, and reactive astrocyte states were interrogated. For better visualization, only results with FDR‐adjusted *p*‐values below 0.05 are displayed in the plot (Figure 5b).

Reactome pathway analysis: Reactome pathway enrichment analysis was performed to identify biological processes associated with genes differentially expressed in the Rq_12_ vs all other conditions. DEGs were obtained from limma analysis and filtered to include genes with an adjusted *p*‐value < 0.05. For the upregulated gene set, genes with log_2_ fold change (logFC) > 0.5 were selected; for the downregulated set, genes with logFC < −0.5 were selected. Enrichment was performed using the enrichPathway() function from the ReactomePA package, using human Reactome annotations and the tested protein‐coding genes in the contrast as the background universe. Significant pathways were defined by an adjusted *p*‐value (Benjamini‐Hochberg) < 0.05.

### 4.8 Western Blot

Proteins were extracted from astrocytes lysed with radioimmunoprecipitation assay (RIPA) buffer *and were* quantified using the Pierce BCA assay (ThermoFisher). Proteins (11 µg/well, boiled 95°C/5 min) were separated through electrophoresis on an 8% polyacrylamide gel in denaturing conditions. Transfer of proteins onto PVDF membranes (Bio‐Rad) was performed for 75 min at 100 V. Membranes were then blocked in 5% BSA in TBST for 1 h at room temperature. Four markers were assayed using the following primary antibodies: POSTN (Abcam, 1:200 dilution), GFAP (Novus Biologicals, 1:1000 dilution), EAAT2 (Santa Cruz Biotechnology, 1:100 dilution), and GAPDH (Santa Cruz Biotechnology, 1:500 dilution). The transfer membrane was incubated overnight in primary antibodies in 2.5% BSA in TBST, followed by HRP‐conjugated secondary antibodies at 1:2500 in 2.5% BSA in TBST for 75 min at room temperature. Bands were revealed using SuperSignal West Pico PLUS chemiluminescent substrate and imaged using Fusion FX7 (Vilber).

### 4.9 Zymography

Conditioned media were collected and centrifuged at 600 g for 10 min to remove cell debris, then subjected to non‐denaturing electrophoresis in an 8% polyacrylamide gel containing 1 mg/mL gelatin. The gel was washed two times in 2.5% Triton‐X for 30 min, then incubated for 24 h in SimplyBlue SafeStain at room temperature, before imaging using a Fusion FX7 (Vilber) imaging device.

### 4.10 Cell DNA Quantification

Cells were lysed in a 0.5% Triton X‐100 and 20 mM ammonium hydroxide (NH_4_OH) buffer. DNA was quantified using the Quant‐iT PicoGreen dsDNA Assay Kit (ThermoFisher), read using a Synergy HT plate reader (Biotek). The number of cells was calculated considering 6 pg of DNA per cell.

Orthotopic implantation and brain collection: Orthotopic implantation of the cells and spheroids was carried out in mice as described previously [127]. This research complies with all relevant ethical research regulations using animals from the Veterinary Office of the Health Department of Canton Basel‐Stadt. Animal handling, surveillance, and experimentation were performed according to the Swiss Federal Veterinary Office (SFVO) guidelines and the Cantonal Veterinary Office (CVO) of Basel‐Stadt, Switzerland. GBM model experiments were executed under licenses #2929_31795. Animals were maintained at the local animal facility in pathogen‐free, ventilated HEPA‐filtered cages under stable housing conditions of 45–65% humidity, a temperature of 21–25°C, and a gradual light cycle from 7 am to 5 pm. Animals were provided standard food and water without restrictions. Mice were anesthetized with 2.5% isoflurane in an induction chamber. Anesthesia was maintained at 1.5% isoflurane delivered through a nose adaptor on the Neurostar stereotactic frame (Neurostar, Tübingen, Germany). A midline incision was made, and a burr hole was drilled at 1 mm posterior to the Bregma and 2 mm lateral from the midline to the right. Cells were dissociated from the spheroids by trypsinization, and stereotactic implantation of the cells (100 000 cells in 4 µL PBS) was performed using a Hamilton 10 µL syringe (#80300, 701N, 26s/2″/2, Hamilton) at a depth of 3 mm below the dura surface. 2 min after injection, the needle was slowly retracted to avoid reflux of the cell suspension. The scalp wound was closed with sutures (5‐0, Polypropylene suture, Ethicon, USA). 12 months later, animals were transcardially perfused with ice‐cold PBS, and brains were dissected, fixed in formalin, and embedded in paraffin for in situ hybridization. Human nuclei were stained by chromogenic in situ hybridization (Zytovision kit) for human Alu repeat sequences following the manufacturer's instructions. Nuclear fast red (Sigma) staining was used as nuclear counterstaining.

### 4.11 Statistical Analysis

Analysis of variance (ANOVA) was applied with the Tukey test, using OriginPro 2023 (OriginLab Corp., Northampton, MA, USA). A comparison between only two values was done using the unpaired *t*‐test in Microsoft Excel (Version 2502, Microsoft Corp., USA). *p*‐values ≤ 0.05 were considered statistically significant. ns = not significant, * *p* < 0.05, ** p < 0.01, *** *p* < 0.001. All values are presented as the mean ± standard deviation of the mean.

## Author Contributions

Conceptualization: V.P.S. Methodology: L.S., T.B., M.S., S.H., R.S., M‐F.R., G.H. Investigation: L.S., T.B., M.S., M‐F.R. Resources: V.P.S., G.H. Visualization: L.S., T.B., M.S., S.H. Supervision: V.P.S., B.H. Writing – original draft: L.S., V.P.S. Writing – review & editing: V.P.S., T.B., M.S., G.H., L.S., R.S., B.H., M‐F.R. Writing – review & editing‐1^st^ revision: V.P.S., T.B., M.S., G.H., R.S. Writing – review & editing 2^nd^ revision: V.P.S., T.B., S.H., G.H.

## Funding

This work was supported by the German Research Foundation (Deutsche Forschungsgemeinschaft) through the excellence initiative of the German Federal and State Government (EXC 294). GH would like to acknowledge the support of the Swiss Cancer Research (KFS‐4382‐02‐2018).

## Conflicts of Interest

The authors declare no conflict of interest.

## Data and Materials Availability

The data that support the findings of this study are available in the supplementary material of this article.

## Supporting information




**Supporting File 1**: advs74138‐sup‐0001‐SuppMat.pdf.

**Supporting File 2**: advs74138‐sup‐0002‐VideoS1.mov.

**Supporting File 3**: advs74138‐sup‐0003‐VideoS2.mp4.
