# Supplementary material for: Stochastic Nanoscale Biophysical Cues as a Basis for the Induction of Glioblastoma‐Like Transcriptional Programs in Astrocytes
Source: Adv Sci (Weinh). 2026 Feb 3;13(20):e09362. doi: 10.1002/advs.202509362 (PMC13067847; doi:10.1002/advs.202509362)
Supplement: Supplementary file 1 — Supporting File 1: advs74138‐sup‐0001‐SuppMat.pdf. [file ADVS-13-e09362-s001.pdf]

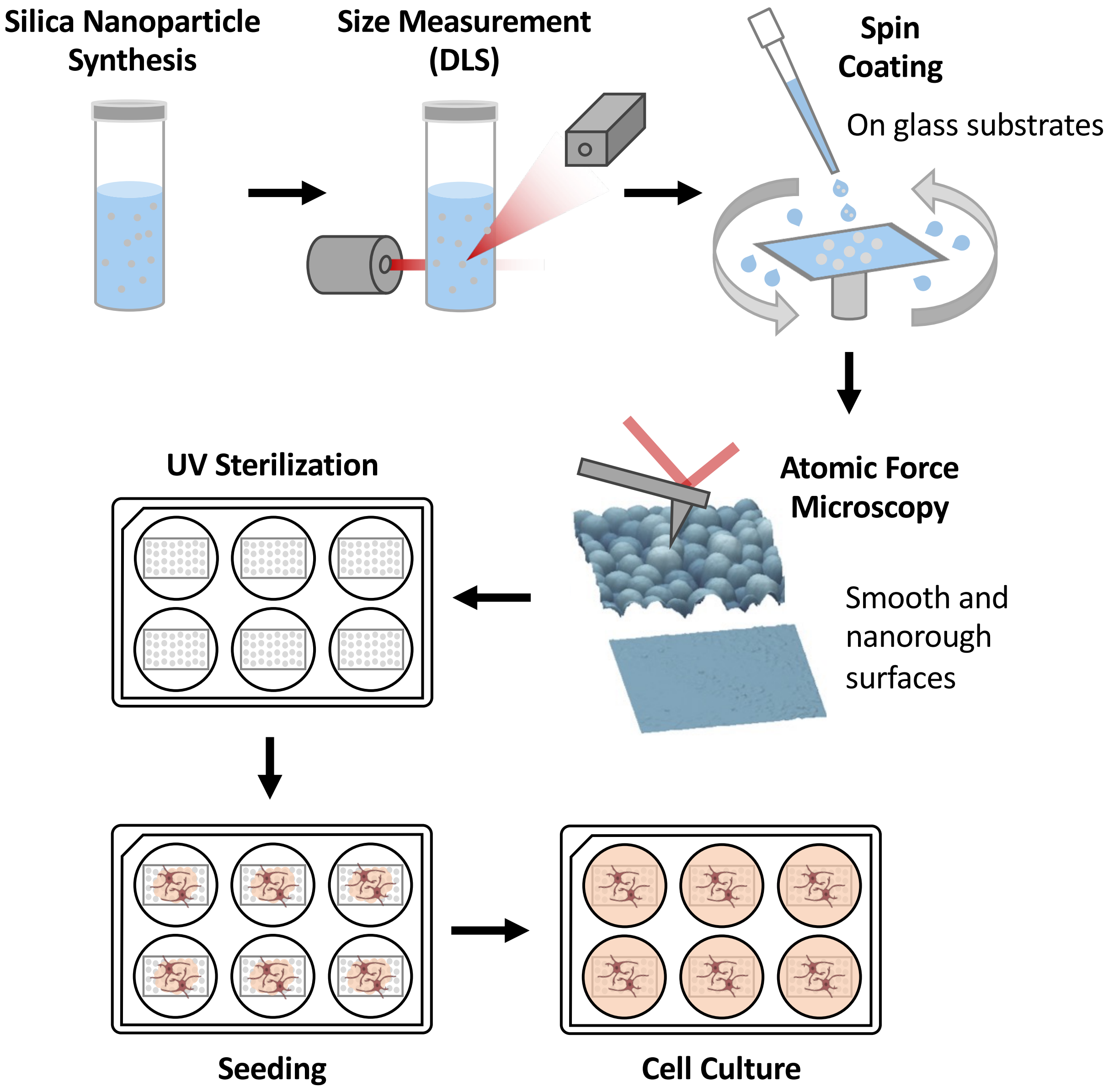

Supplementary Figure 1. Experimental design. Silica nanoparticles of various sizes were synthesized using the Stöber process, measured using dynamic light scattering (DLS), and spin-coated on glass substrates. The stochastic nanoroughness (Rq) was measured using atomic force microscopy (AFM). Coated substrates were sterilized by UV light before cell culture. After the initial seeding of 30 min, 2 mL of culture medium was added.

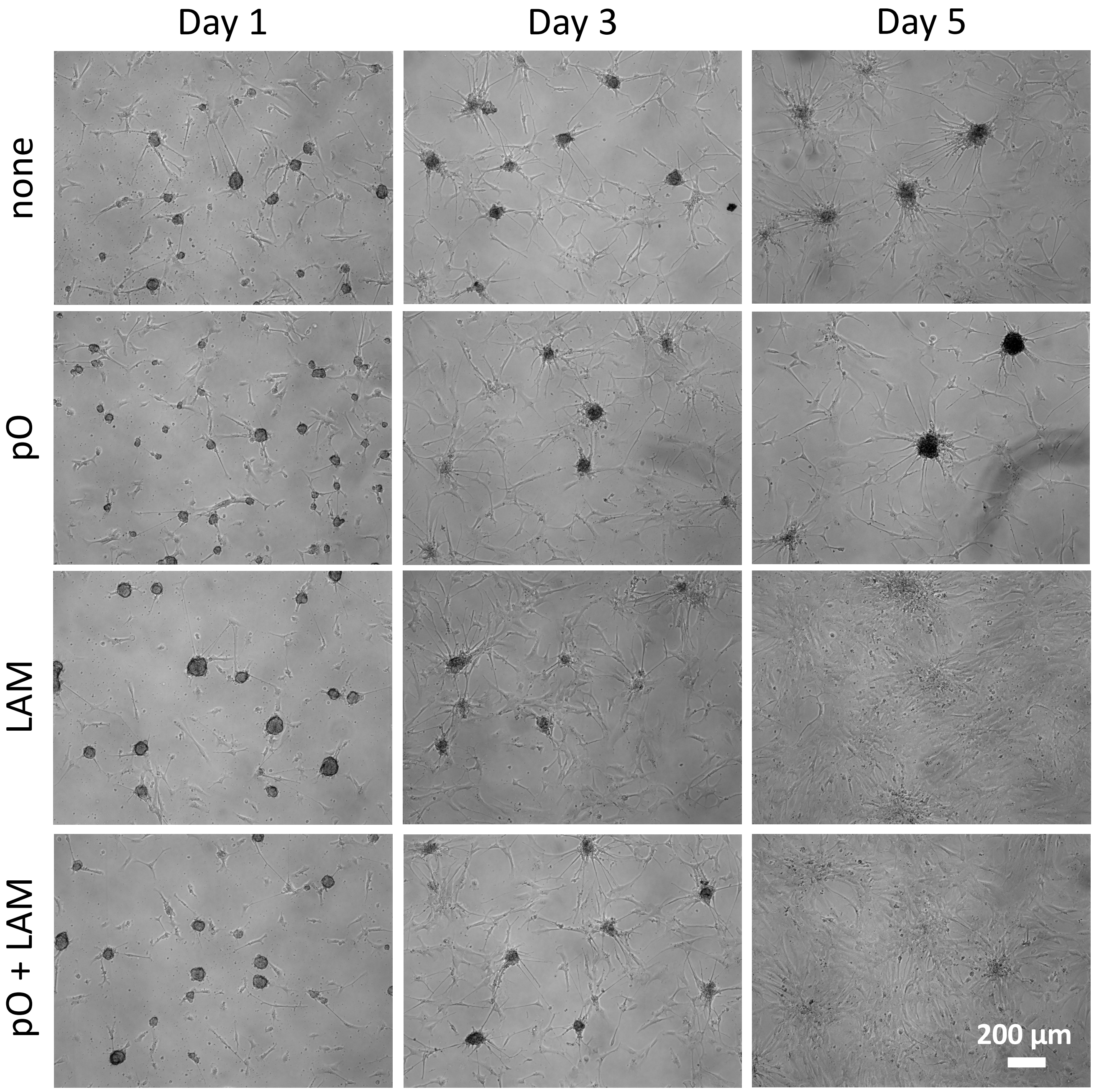

Supplementary Figure 2. Surface coating and spheroid formation: Astrocyte culture on Rq_{12} substrates over time with different coatings of poly-ornithine and laminin. pO – poly-ornithine, LAM – laminin, none – no coating.

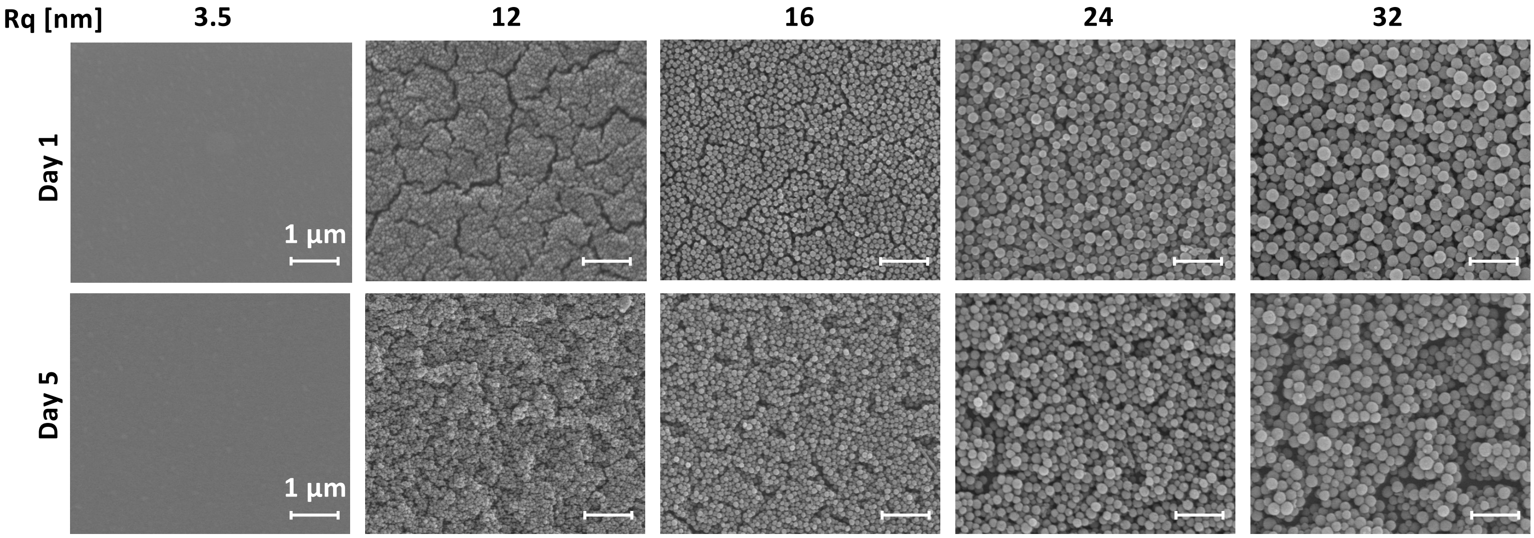

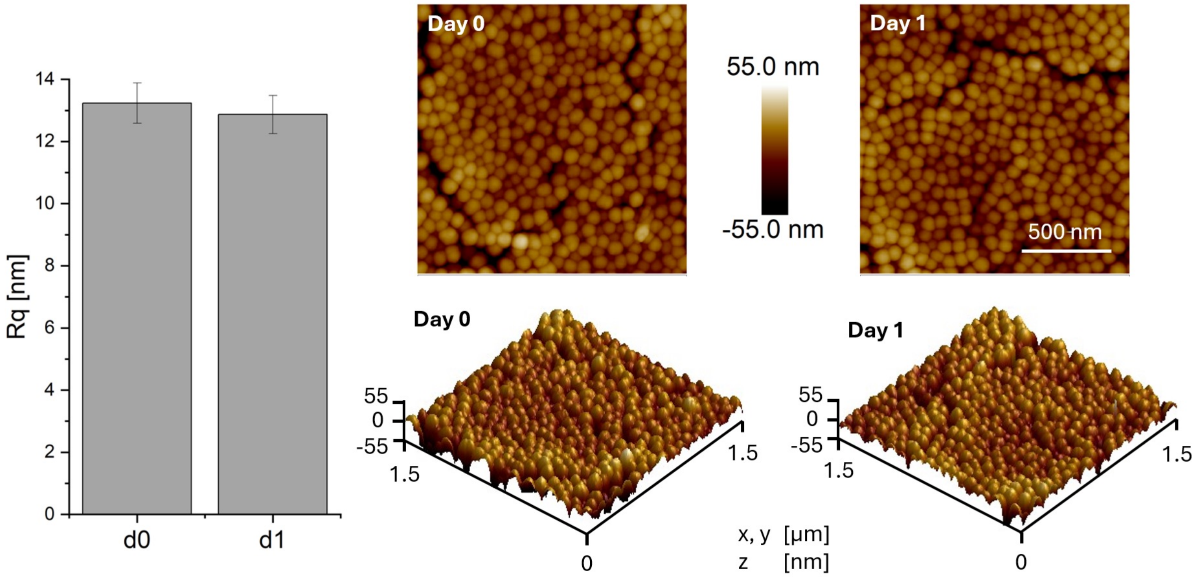

Supplementary Figure 3. Stability of substrates: Nanorough substrates remain stable under the cell culture conditions, at 37°C throughout the spheroid formation. **Top panel:** SEM images of the surface. **Bottom panel:** AFM scans (top: scan images, and bottom: projection image) of Rq_{12} substrate on day zero before incubation in culture media, and after 24 hours incubation in culture media. The graphs show the average Rq values from three different regions on the surface.

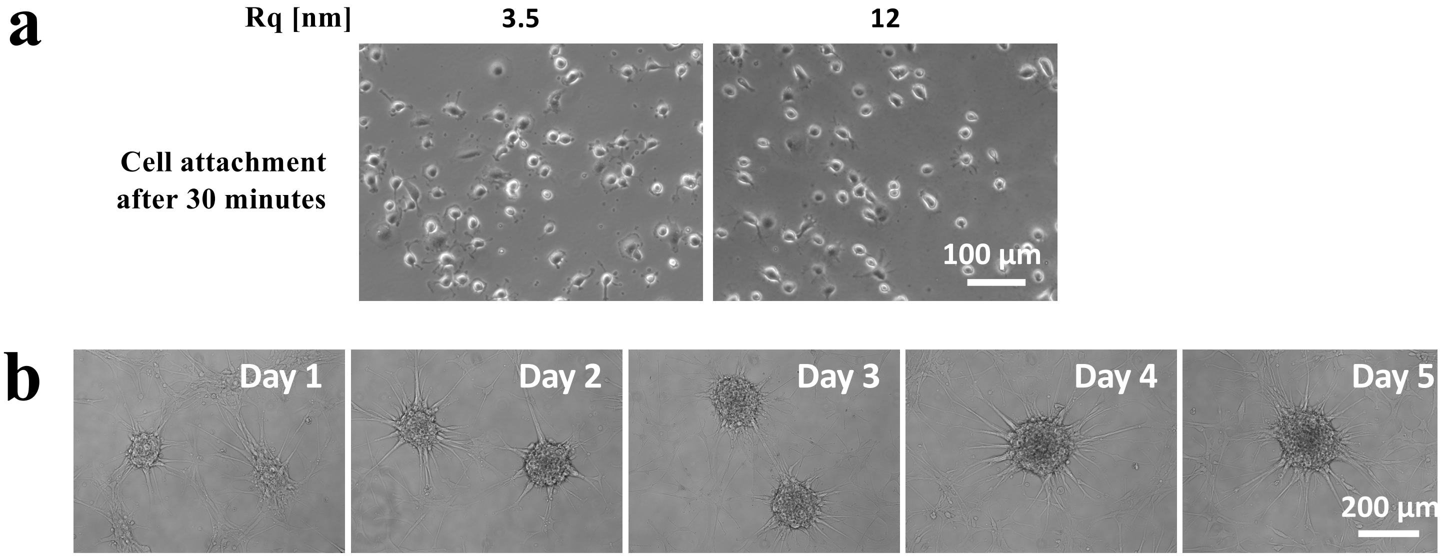

Supplementary Figure 4. Astrocyte attachment and time course of spheroid formation: (a) Optical micrographs showing similarity in the initial attachment of astrocyte on glass and Rq_{12} is not impacted by nanoroughness. Astrocytes were imaged 30 minutes after seeding. **(b)** Representative images of spheroids on an Rq_{12} over 5 days.

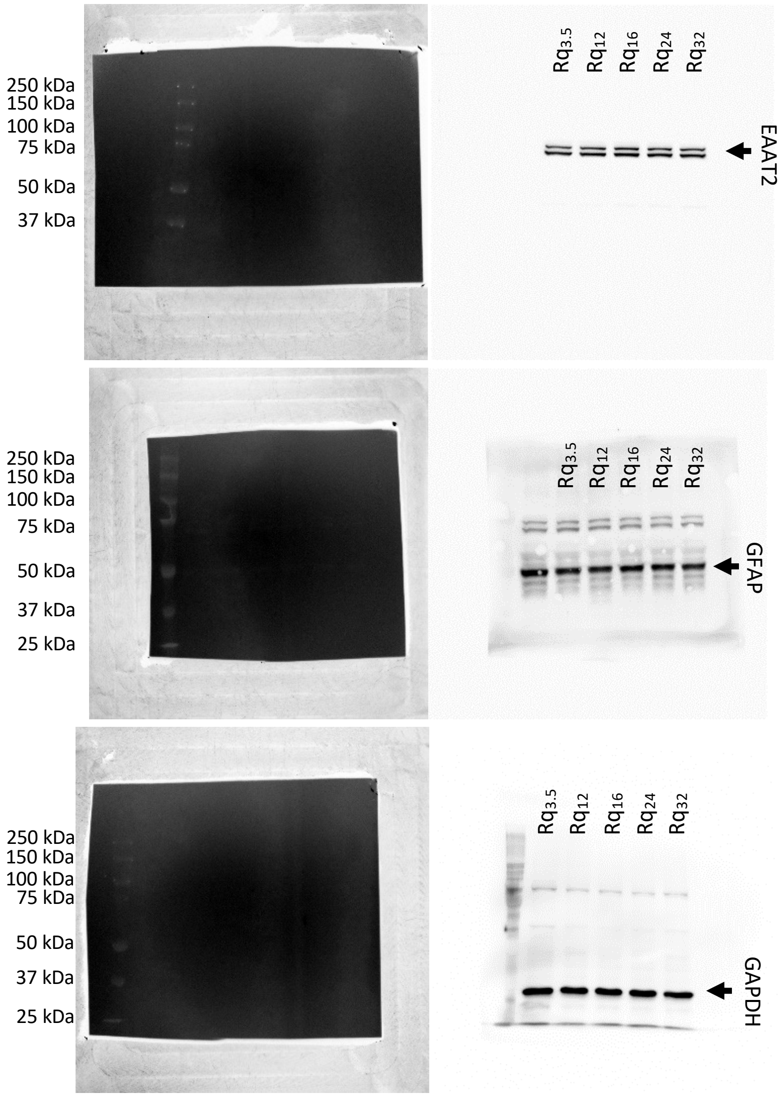

Supplementary Figure 5. **Full Western Blots of EAAT2, GFAP and GAPDH in Figure 1g.** Ladders with protein sizes indicated. EAAT2: excitatory amino acid transporter 2, GFAP: glial fibrillary acidic protein, GAPDH: Glyceraldehyde-3-phosphate-dehydrogenase.

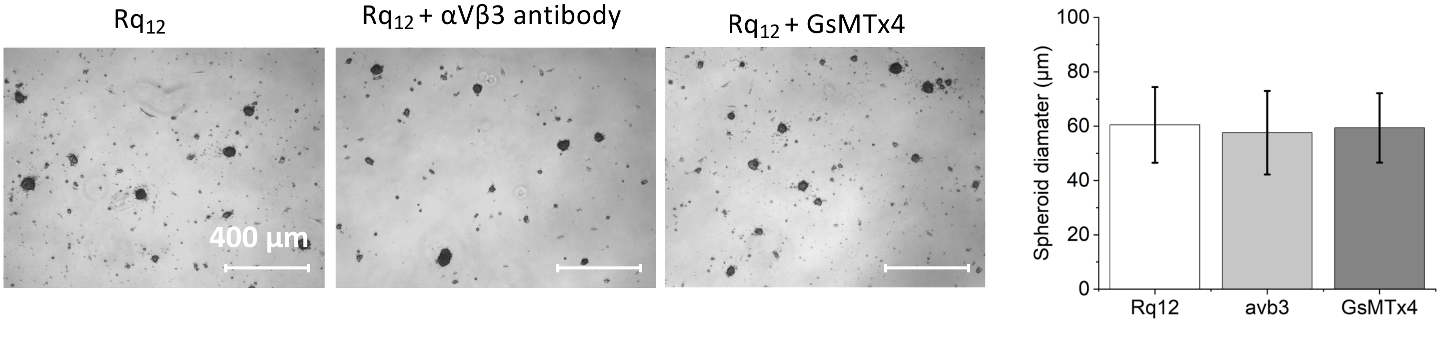

Supplementary Figure 6. **Effect of integrin and Piezo-1 pathway on spheroid formation:** Blocking Integrin αVβ3 using a blocking antibody, or blocking the piezo1 receptor using the spider venom toxin GsMTx4, does not impair spheroid formation on Rq_{12}.

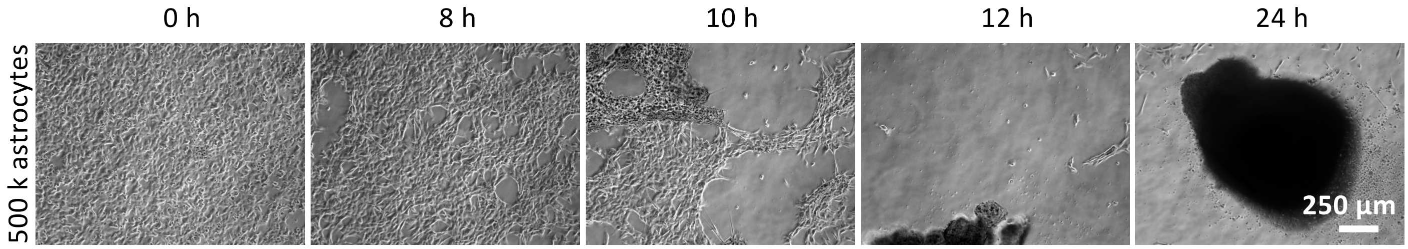

Supplementary Figure 7. Spheroid formation at high plating density: Formation of spheroids on Rq_{12} over 24 h at a plating density of 500k cells. Detachment of cells and spheroid formation starts as early as 10 h.

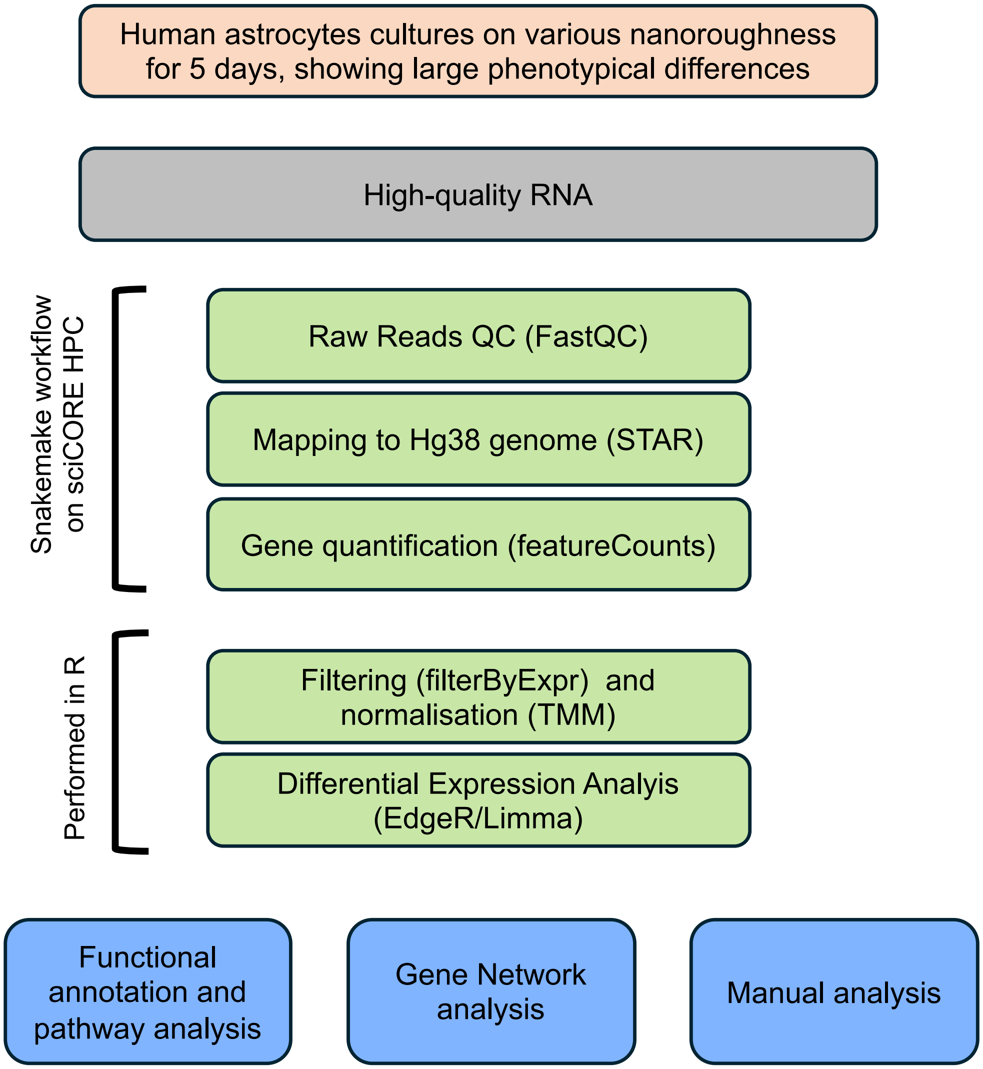

Supplementary Figure 8. Overview of the RNA-Seq computational analysis pipeline.

UCN2	TLCD2	BNC1	TGFB1I1	PIM1	ACCS	FGFR4	NEIL1	DNAJC6	ENSG00000271254	CDK11B	CPT1B
TNS2	COL27A1	PGM2L1	NHSL1	SYNE2	GAPDHS	ACIN1	LAYN	RABL2A	PLA2G4B	KLHL8	MFSD10
COL6A1	RECQL5	COL6A2	AGAP5	FUS	CEP164	SEMA4G	MATCAP1	CDK2AP1	LBH	RDH5	NCS1
IZUMO4	GALNT16	ZNF692	MT−ND3	FTCD	ZMIZ2	HIPK3	QPCTL	EIF5A2	OSBPL7	YPEL2	PHYKPL
PILRB	RGS4	SULT1A3	HDAC7	MAGED2	TNKS1BP1	ENPP4	SLC5A3	ANKRD40	SIDT1	DLC1	GLIPR1
MT−ND2	CDK5R1	CCDC78	YAF2	ZBTB20	MRI1	LCNL1	FSD1L	BACH2	MIER3	TMEM249	RASA2
TOB1	TUBE1	PILRA	KSR1	CNOT3	ATP6V1C1	SMARCC2	NPIPB12	ANKS6	ARMCX2	D2HGDH	ELFN2
MST1	SOX6	DDX39B	GGH	ZFTA	TMEM165	NAP1L3	NAB2	SIRT1	FUCA2	IRF3	KLHL25
COL7A1	QPCT	ARHGEF25	PCSK4	SPRING1	LSS	PIH1D2	SREK1IP1	FAM193B	SPEG	CLBA1	ZNF284
GGT5	PIAS3	PTMA	PLEKHG5	LUC7L	YWHAQ	ARAP3	ENSG00000288645	MUSTN1	MMS19	MAGED4	SOST
ITGA7	TEPSIN	PLEKHF2	HDAC6	TYK2	ZMYM3	LAMA5	SUSD2	RNF13	SREBF2	PCED1A	ZNF675
EML2	BTG2	NUDT17	CDKN2B	SMARCD3	TSPAN12	KIFC2	GAREM2	COMMD7	TMX4	CHKB	WNT7B
WDR97	ADAMTS15	ADGRL1	PCOLCE	GRIPAP1	SERPINI1	LTB4R2	CPEB4	RAG1	SNRNP70	GPSM3	CCDC102B
ASPN	MICALL2	OPLAH	ARSA	RHBDL1	FAM169A	ZNF615	LZTR1	NUB1	ENSG00000285382	FST	ATP2A1
NFKBIZ	MZF1	VARS2	CPSF1	ARL5B	ENSG00000288716	SOCS5	INSIG1	CYP27A1	MOAP1	TBC1D3I	AAAS
SLC25A37	CLTCL1	ENSG00000288000	NXF1	EHD3	TBC1D3K	CCDC188	PRPH2	SPAST	KLHL11	CNOT6L	CDSN
ZC3H12A	KCNG1	TUBGCP6	PURA	DSCC1	MINK1	ARMC10	ARID5A	CACNA1B	POMT1	CALCOCO2	ENO2
APOL1	TAF15	PRRT2	FGF5	TRIM2	GLI1	C21orf58	ENOPH1	NOTCH3	RSKR	SULT1A1	POFUT2
LRRN1	LRRC8C	TIMP3	LRRC4	DENND5B	XPC	DDI2	GRK5	DENND2C	CSF2	PODN	NTM
RHBDF2	BTBD19	MAN2C1	CYP2U1	SLC9A5	CDK18	AP1S3	SLC25A46	PCGF6	DXO	NOMO2	ENSG00000288637
GULP1	RBM5	LUM	NBEAL2	TNFRSF25	AKAP8L	TPCN1	PJA1	INCA1	GSKIP	USP4	DEPP1
MEX3A	HSPA5	STRA6	PML	LRCH4	NISCH	ABRACL	ZNF891	TTC31	SLC11A2	SEC24C	NAB1
RAPGEFL1	TAF1C	LPCAT4	CDK11A	TMEM80	ZBTB41	ENSG00000290315	PYROXD2	SCNN1D	POU2F2	C8orf44−SGK3	NUFIP2
ADAMTS10	ENSG00000250264	MAEL	MYOM1	KBTBD8	KLHL15	PSMB10	PKD1	BMP2K	CASP3	INHBB	FASN
FLVCR2	VILL	LAT	ANPEP	SEL1L3	DNAJB9	NOMO1	MAST2	TBX15	ZDHHC21	TMEM175	LAS1L
PAN2	BEX2	ACTMAP	YARS1	SERPINH1	SGSM2	PCLO	TRUB1	PTGIR	PTTG1IP	IL6R	PPIL2
ADGRA2	SUGP2	SHMT2	MVD	SESN2	TP53	RNF11	CYLD	MSC	PKIA	TRPC4AP	CCDC69
BGN	SLC35G2	CD58	CCS	YPEL5	FDFT1	HMCN1	PRTG	ADAMTSL1	CASTOR2	ZNF431	SLC36A4
GIGYF1	NFKBIA	PLXNA3	TSSK3	KDM4B	BMI1	IFT140	NPIPB15	SOCS6	IRS1	GOLGA7	DPYSL5
NEURL1B	CX3CL1	BTN3A1	KLF7	MAP4	AIRE	BTN3A2	BHLHE40	UBA1	MED15	ZNF529	RHOT2
ZNF460	MOV10L1	PIDD1	LGALS3BP	STK38L	LTB4R	NCKAP5L	FCHSD1	VPS9D1	PAQR3	RCN3	MVK
KAT2A	PDE8B	EPSTI1	ATXN2L	ABHD17B	NLGN2	POLM	ADCY4	ARFGEF3	ZBTB34	HECA	MSANTD7
NPIPA9	CDK5RAP3	DSG2	PDGFD	MDC1	PGAM4	PYGO1	USP21	ZDHHC2	GPATCH11	VIM	TTYH2
TRPM4	WDR90	DUSP7	ADCY6	GGA1	ZDHHC8	GSDMD	SPACA6	FAM83G	SPRED1	MAGED4B	C19orf44
DENND1B	MAGED1	NEURL4	ZNF79	ALDH1L2	CDK10	ITGA6	TMCC2	C9orf72	OSBPL8	PAQR6	TPD52L1
PDGFRB	FAM200A	AARS1	SARS1	PRR22	CADPS	ZCCHC2	ZFP14	SALL4	FGF13	EMX2	PCDHB14
CDK3	MMAB	CIRBP	VCPIP1	S100A11	CCR7	B3GNT2	DAAM2	NCEH1	NPIPA2	PER1	
RBM33	GPSM1	EPRS1	GANAB	TCIRG1	GALT	IFT172	NAA30	COL11A2	COASY	MAPK11	
HDAC10	ADAMTS14	KCNIP2	SRSF5	WDR17	FZD3	NAPEPLD	CXCR5	GGA3	TMEM60	BLTP3A	
EPAS1	RFK	SLC12A4	HSF4	HMGB1	SRSF6	STAT2	ECE1	ABCA13	SLC15A4	CDYL2	
B3GALNT1	NES	AMH	CALML6	MYO5B	SULT4A1	MEOX1	ZMPSTE24	CAB39	ZNF783	TEAD3	
FBLN2	STARD13	TRO	SAMD9L	MT1F	PPTC7	STK11IP	SPDYE2B	CFAP44	TRIP10	ADGRF3	
INTS3	GPR173	GADD45G	SKIC2	ZNF823	ZNRF2	ZNF792	TMEM169	PM20D2	DELE1	TP53I11	
EPHA5	FAM107B	SLC39A10	EHD2	CC2D1A	DRD4	ITGA11	SPTBN5	CCHCR1	CCNG2	NET1	

Supplementary Figure 9. Full list of the 520 DEGs specific to astrocytes grown on Rq_{12}.

CFI	LRRN3	CADM1	TRDC	PIPOX	CCND1	GMPR	COL4A6	RASSF10	NRG1	PTCHD4	TRIM58
SLC2A12	MGLL	SOX2	SEPTIN4	STON1−GTF2A1L	MFAP5	PCED1B	TGFB3	DCLK1	MT2A	SLC34A2	HMGA1
GDF6	PLEKHG1	CXCR4	TRIB2	OLFM2	PIANP	PTN	ANOS1	CPED1	SHROOM3	ARHGAP22	CHRDL1
MARCHF4	LRATD2	SELENBP1	GPC4	ADD2	ENSG00000285330	SVIL	VSIG10L2	PBX1	MYO5C	COL24A1	
GJB2	STON1	AOX1	CDON	LIMCH1	CPZ	JAM2	EFNA1	SLC7A2	MOXD1	ENTPD2	

Supplementary Figure 10: Full list of the 58 DEGs specific to astrocytes grown on Rq_{32}.

CEBPB	L1CAM	CALCRL	TM6SF1	NEK6	ANKRD9	ATP2B2	ADAT2	PLA2G3	LOX	NPC2	ABCA12
RASL10B	MYO1F	TWIST1	BAMBI	TNNT1	PLAAT5	ANTXR1	ADAMTS6	DPP7	TUSC1	CRB2	PTP4A3
EPHB3	ENSG00000287856	LRRC17	IL6	LRP1B	SMAD6	CTSO	SULT1B1	METRN	QPRT	FAM111A	AHCYL2
ADAM22	C11orf96	ENSG00000286190	CKLF	TSPAN7	NUDT14	SLC6A8	INSYN2A	IQCE	RPE65	ALDH1A3	NEIL3
EBPL	CEACAM19	PLEKHB1	AHRR	SPX	TXLNB	CTPS1	TMEM59L	PLN	TPST1	PROM1	PSEN2
ENSG00000293584	COL10A1	LY6K	ELMO3	EMID1	SETD6	COPRS	FRMPD3	CASP7	SCN9A	CHST1	A4GALT

Supplementary Figure 11. Full list of the 72 DEGs specific to astrocytes grown on Rq_{16}.

SPOCD1	SLC24A2	GCNT4	RPS6	FRZB	EPS8L2	CACNA1A	RPL38	RASSF9	KDELR3	FGFR1	TMEM100
DKK2	MGARP	DIRAS3	SPRY1	RPL23	HIP1R	TUFT1	ALDH18A1	NPC1	LACTB2	EEIG2	NSMAF
ADAM12	WNT2B	KITLG	HS6ST1	RPS8	C1orf198	TNIK	ZDHHC9	CD200	GRAP	LONRF2	MYH10
ITGB4	C10orf90	AJUBA	UNC5B	SDCBP	EPB41L2	MGAT1	SAMD4B	SFRP1	AADAT	AEBP1	FNBP1
NFASC	VDR	RPS3	PRRX1	ITGB6	COL6A3	GPR158	CHRNB1	PCDHA6	OSBPL1A	SEMA3B	INPP5F
RGL1	EMP1	PCDHA10	INSYN2B	AMDHD2	EFR3B	PNMA2	RADX	RPL13A	FMNL3	AHR	ENSG00000271810
GPRC5A	CNN1	CD93	ARHGAP20	SPINT2	ZBTB46	PCDHA5	GRIA1	GSTM3	PALM	TNNC1	ARSJ
FGF7	CTSK	PCDH17	TNFRSF6B	CEMIP	SYNPO2	RPL3	SLC47A1	PPP1R3B	PMP2	LTA4H	RPL27A
EFEMP1	RASL12	EFEMP2	HOXB6	SLC2A13	OXTR	BEND6	SELENOW	ATP6V1A	KCNT2	PIWIL4	KLRC3
FAP	COPZ2	ZNF175	SHC4	NEFL	HNF1B	GBX2	SMIM3	STXBP5	ALDH1A1	CD44	RPL21
LIPG	TBC1D8	OSGIN1	CLIP2	EFNA5	MAPK10	CAVIN4	ZNF365	SPCS3	RPS27A	ZFYVE26	SKAP2
ADGRL4	ALPL	GNG2	PLEKHA2	CELF6	RPL32	SPART	ITM2C	PBX3	RPL27	F10	NFE2L3
TGFA	INAVA	RGS17	LRRN2	ANKRD37	ADAM19	NFATC2	PCDHA8	CAVIN1	NRN1	KCNK2	PALD1
C5orf46	RPS29	KIF1A	TCIM	CORO2A	SLC4A8	RASSF7	ACVR1	CYTH3	NETO2	TUBB6	FANCF
ITGA5	IGFBP7	ZNF395	ILDR2	PCDHB16	AJAP1	RRAS	PRDX4	PKN3	VASH1	CTNS	HPS3
APOD	SLC9A7	LYSMD4	RNF157	EDIL3	CLN8	ODAPH	STARD8	NIPAL2	ESR2	CCDC28B	BCL2
SOWAHB	RPLP0	DOK6	SIK1	CARD6	RPL14	APOLD1	SALL1	RFTN1	TNIP1	GALNT1	GRN
HIVEP3	PPARGC1B	DOCK2	ST6GAL1	ENSG00000251569	HSD17B2	KCNA2	RPL10	CRHBP	FLT1	GGT1	RAB9B
RPSA	ENSG00000248235	VASH2	TEK	HBEGF	BHMT2	NIPSNAP1	NUAK1	SELENOM	DCXR	NECTIN1	XDH
HRH2	VWF	ARRDC4	LAMB3	NOVA2	SYNGR3	APLN	DNM1	MAP3K14	SLC25A1	SATB1	SRA1
COMP	LURAP1L	PTHLH	WASHC5	MISP	TIMP2	QSOX1	MYOM3	UCP2	RHOC	SLC36A1	GNA14
PLEKHA4	CTNNAL1	RPL4	IGDCC4	TRHDE	KCTD20	G0S2	RPL23A	HMGB2	PRELID2	CSF1R	ADGRL2
DOCK10	SORL1	MDFIC	TGFBI	CA9	KY	ARHGAP23	SLC13A5	RGS10	TDRP	ERCC6	TEX9
KCNK6	KCNK3	JARID2	KCTD16	ANK2	HMSD	RPL18A	TMEM256	CHN2	MTURN	GAS6	SMAD3
OLAH	AKAP12	GATA6	ADGRB2	POSTN	GDF5	PLA2G15	NKAIN2	C3	LTBP2	FABP3	
PRKG1	EPOP	REXO2	SQOR	FMNL2	ACTG1	SLC7A11	PNMA1	LRP8	CASTOR1	GARRE1	
TMT1B	ELK3	ADAMTS3	SH3KBP1	HRCT1	PIK3R3	NDNF	PHACTR1	KHDRBS3	MROH1	PACSIN3	
ZP1	GCLM	COBLL1	SNX8	DERL3	HOXB3	PDE3A	RPS16	CPA4	EBF1	SERPINA1	
SUSD6	MARCHF3	RPL5	SLC37A1	EEF1G	BMPR1B	LGMN	ATP8B2	FBN2	BFSP1	NLRC5	
CLIC3	SLC16A3	PTPRF	AFAP1	EPS8L1	AFF2	EPHA2	RFX8	PTPRR	ADAM23	RACK1	
FER1L6	SLC41A2	EFR3A	HMGXB3	TENT5A	ACKR3	WDR1	EEF1A1	PCDHA9	STEAP1	RPGR	
MFAP3L	CDH5	ANO1	EEF1B2	NXN	NANS	RPS12	TOM1L2	PLAAT4	NRG3	GLIPR2	
RPSA2	RPL26	PKP2	LACC1	NRXN3	PFKFB2	MYH15	VKORC1	NCR3LG1	HOMER2	BDNF	

Supplementary Figure 12. Full list of the 387 DEGs specific to astrocytes grown on Rq_{24}.

MT−ATP6	NPIPA1	ARHGAP33	PLEKHG4	TLR3	BTN3A3	FBXO33	NPIPA5	HEMK1	FNDC4	FAM210B	ZNF503
MT−CO2	XAF1	TRIB3	SLC26A6	CCDC126	SHC3	NICN1	SLC44A5	DTX1	FAM13C	ATF3	KALRN
MT−CO1	LENG8	NPIPA7	PKNOX2	PLEKHG2	ANKRD6	SP140L	PRXL2C	ERF	CES1	CCDC9B	SLC22A4
MT−ATP8	PRUNE2	MAMDC4	VEGFA	LIME1	NEDD9	SERPINE1	LENG9	PSME2	PIF1	RNF145	CKLF−CMTM1
MT−ND4	ENSG00000183889	TTLL3	ITGA10	PABPC1L	ATAD3C	CCNL2	ITGBL1	DOK3	TBC1D2	HNRNPH1	FGF14
MT−ND5	TSPYL2	ROBO3	ODF2	LAMB1	DGKA	ID1	TARS1	GOT1	PLPP2	EMILIN3	LDLRAD2
AMT	GOLGA8B	PRSS53	COL1A1	PNISR	LTBP1	PSMB9	NDFIP1	CERK	KRTAP2−3	TUBG2	PROSER3
MT−ND6	SEC31B	PRICKLE4	NRBP2	EGFL8	APOL2	GSDMB	PDLIM7	CNTNAP2	GPRC5C	DACT3	
MT−CO3	LCAT	ARHGEF2	NID2	GNB3	SEPTIN6	ZNF184	ATP6V1G1	GNAI1	TTC7A	RAB22A	
MT−CYB	MAPK8IP3	GTPBP2	APBB3	ARHGAP26	RBCK1	BAG1	B3GNT7	ZNF709	LAMA3	ZNF487	
MT−ND1	AP1G2	LY6G5B	CSAD	NAT9	PNN	ACAD11	DMPK	FAM181B	TIPARP	HOMER3	
UBA7	SPPL2B	MICAL1	CAMTA2	APOL6	IARS1	IQGAP3	THAP10	NPPB	TMEM178B	TRIM22	
MT−ND4L	COL1A2	RBM6	MEGF9	CDH13	NPIPB4	LHX2	MEX3D	ENSG00000282988	HNRNPA1	PHLDA1	
COL5A3	LAMB2	GABBR1	TAP2	ELN	LEFTY2	HOPX	INSYN1	ENSG00000196826	THRA	CLK2	

Supplementary Figure 13: Full list of 161 DEGs common to astrocytes grown on Rq_{12} and Rq_{16}, versus smooth

SPP1	KIAA1755	GABRE	SIGLEC15	NAV2	KCNJ15	TNC	CDCP1	LOXL4	NKD2	TMEM158	NPTX1
A2M	HSPA6	TNFAIP8L3	TLL1	IFI30	FZD8	DKK1	SH2D5	ACTA2	LYVE1	SHISA2	RASSF4
GPNMB	SLC16A6	VCAM1	SEMA7A	VAMP1	DGKI	HMGA2	AHNAK2	SLCO2A1	ENSG00000289697	LDLRAD4	ENC1
CHRM3	IBSP	C5AR2	GRIN2A	ITPR3	GPR68	LAMA1	CCL2	FGF11	CFH	ELFN1	

Supplementary Figure 14. Full list of 47 DEGs common to astrocytes grown on all conditions (Rq_{12}, Rq_{16}, Rq_{24}, and Rq_{32}) versus smooth.

TMEM132B	PDPN	MMP14	LRRTM2	DNM3	MBP	CDC42EP2	UACA	TMEM35A	NRROS	COL8A2	TMEM171
MMP11	SLC19A2	RAB27B	CCN1	PROS1	RASGRP2	P4HA1	TBX3	SPHK1	C7	GPRIN3	DTX4
ANGPTL2	APCDD1L	ALDOC	PTGDS	OGT	DMD	SQSTM1	PROX1	EDARADD	PRKAR2B	CTSD	CCDC71L
CDH23	INHBE	MAF	CYP1A1	HEYL	RUSC2	PPP1R13L	CNTNAP1	CD9	CCDC80	DUSP2	XKR4
GEM	SLC17A5	SGCD	KCNN4	EHBP1L1	RALA	STRIP2	CPVL	SIDT2	TYRO3	TNFAIP6	NTN4
MEDAG	ITGA8	SLC7A8	TNFAIP2	SIRPA	FADS2	CERS4	MGST1	CSMD2	SLC44A2	IFITM3	KLF11
CCN3	DLK1	LMO2	GAP43	B3GALT1	P4HA2	IGSF9B	DENND2A	SLC1A4	ST3GAL1	GBA1	TCAF2
NKD1	PHGDH	ABHD2	NKAIN3	CRACDL	ELOVL4	INPP4B	MDGA1	TPM1	SCARB2	PLEKHM1	CNN2
COL14A1	B4GALNT3	ADCY8	SOD2	HEY1	COL4A5	TMEFF1	IMPDH2	ITGB5	NDST4	ADGRE2	PDLIM1
CRYAB	CCDC3	SYNM	DYRK3	SYT15B	C6orf15	GALNT15	TM4SF19	PPARG	NR1D2	CYSTM1	FILIP1L
BEAN1	SLC6A1	LAMC3	HKDC1	ADAMTS16	KLHDC7B	MMP13	ANXA8	DUSP3	ETV1	IGFL3	TMEM192
BMF	KLHL24	ENSG00000283189	FHL1	AJM1	CLU	SREBF1	SCN3B	C6orf132	CHMP1B	KLHL41	GAPDH
CYP1B1	COL5A2	COL5A1	TRIM62	E2F7	DIRAS1	CAMK2D	MFHAS1	NFIL3	AKR1C3	HSPA2	SNTB1
MTSS1	MCHR1	SORBS2	ENPP5	CRISPLD1	MMP15	MSANTD3−TMEFF1	ZNF697	CRTAC1	PDLIM2	PCDHB10	COL22A1
KANK4	PLXDC1	SLC4A10	S100A10	GRIA3	ACSS2	B4GALT5	CD68	CXCL12	MYO1D	C5AR1	GJB3
NDUFA4L2	HDAC9	CHI3L1	CPE	EDNRB	ARTN	FBXO32	DES	ZFP36L1	FHL2	STOX2	TNFRSF21
HMOX1	F13A1	FNIP2	FNDC1	GLCCI1	NEU1	PYCR1	CADM3	LRRC32	RAB7A	PDE1B	TRIM9
ASNS	FAM156B	WARS1	HS2ST1	ADAMTS12	CLDN4	OSBP2	TMEM255A	CHST3	JPH3	APBA1	NRCAM
GPM6A	SLC17A9	ANO4	NPTXR	DHRS3	AK4	CTSB	ANKRD10	NHERF1	SMAD7	MYLK	IFNGR2
CYP26B1	PKP1	CBS	GARS1	ENSG00000267561	CXCL8	KDR	NR3C2	FLT3LG	ENSG00000250644	STK38	KLF13
GUCY1A2	CHAC1	KIF26B	ESM1	GDF1	WEE1	GRIK2	VSTM4	RCAN1	SYPL2	LIMS2	GPR137B
RETREG1	RRAGD	FNDC5	NR4A2	TIMP4	PTGER2	FKBP11	ITGA1	PNOC	ST3GAL5	TPI1	SHISAL1
GOLGA8A	PGGHG	SYT7	ANKZF1	SPON2	GYS1	SCD	TINAGL1	CPNE8	PRSS23	ENSG00000285043	VAT1
APOL3	KCNS3	PCSK9	PPFIA4	HS3ST3B1	MEGF6	PLIN2	LOXL3	CHST11	TMEM117	GPM6B	SLC35F1
LPIN3	COL21A1	MYC	BMP1	SLC37A2	SNX9	ENPP1	SIX2	CMKLR2	HPCAL1	GLIS2	UAP1L1
RNF128	COL3A1	P4HA3	THBS2	STEAP3	CLEC18B	DYSF	TENM4	FOXF2	EDN1	SERPINE2	DPEP1
PCK2	STX3	ASAH1	TGM2	RRBP1	TMC7	CLMP	SCG2	PNP	GM2A	PTGES	CLEC18C
IRF1	PLXNA4	ERG	BEGAIN	PGK1	CARS1	TUBA4A	LMO7	ADGRA3	IRAK1	PDK4	PGAP4
RDH10	TSPAN13	GASK1B	SPARC	TCEAL7	RRAGC	LDHA	CRACD	DNAJA4	SORBS3	KCTD12	SSC5D
HCN1	IL11	MYRF	PPP1R14C	CDKL2	SOAT1	SPOCK2	HEXB	FOXRED2	GREM1	VGLL4	VLDLR
XYLT1	SCARA3	DUSP10	SLC1A5	GPT2	EBI3	FLG	FADS1	COL9A2	C1QL2	TTC39C	LARGE1
CD24	MCUB	SYT12	FGF1	KLHL13	ADGRG1	NUAK2	LDLR	CFAP54	C1S	CERT1	TTYH1
MGP	EEIG1	EDNRA	ITGB3	STARD10	GPAM	SLC20A1	SNAI1	IL20RB	MAPK8IP2	KDSR	FOXQ1
IL33	PCDH10	RNF144A	PSPH	PPP1R3C	RAMP1	ADAMTSL2	LPCAT1	DUSP4	BNIP3	NDRG2	SPG21
MEF2C	AP1S2	PDGFA	CPEB1	PLPP3	SLC22A3	RASL11B	B3GALT2	MTHFD2	ECSCR	NKAIN1	AVPI1
IGFBP5	SYT15	PSAT1	CAMK2N1	RGS2	DYRK2	GCLC	UBL3	SLCO3A1	RAB33A	LRATD1	MN1
PTGS1	SLC6A9	TCF7	CREB3L1	THAP2	RAB29	CTIF	GBP1	SIX1	METTL9	M6PR	IGF2
PFKFB4	CLEC2B	MAN1C1	GNPDA1	UBE2D1	MRNIP	NCALD	ACSL1	GPR153	SOX8	PLEKHG3	IL1R1
XIRP1	PLVAP	FNIP1	TMEM200C	RASSF8	ZMYND8	TPPP3	MANBA	C8orf58	MBOAT2	BEST1	SHC2
PTPRN	SYT14	ENSG00000265118	CA12	POPDC3	KLRC2	DTNA	TMEM38B	KIF5C	RASEF	ARHGAP24	IRAK2
MMP7	KRT8	EPHB1	CD109	PHYHIPL	PHYH	NAT8L	B3GNT5	ARHGEF16	TSPAN11	COLGALT2	SOX4
CXCL1	CTH	EPHA7	CAVIN2	PRXL2A	FPR3	ARHGAP9	MID1	CTSL	BEX4	BPI	STK32A
HSPB7	LYST	PYGB	PRR15	SNX30	GLT8D2	SPAG4	ERMN	HIPK2	ATP6V1B2	HSD17B14	TMEM119
MCTP1	MXRA5	COL4A2	ID2	KCNMA1	COL8A1	ASS1	PCDHB9	SCG5	SDC1	SUSD4	SPARCL1
CRLF1	CCN2	MEF2A	GPRC5B	TXNIP	MTHFD1L	SLFNL1	SLC24A3	RUNX1	CREG1	PCSK1	ADPRHL1
LSAMP	SYNPO	BCL2L11	ZCCHC14	RENBP	DCT	ITGA2	LRRC8A	UNC13C	PSME1	PGBD5	GAL
ADM2	FIBCD1	FAM156A	COL4A1	LRRC15	ZNF521	DPP4	FAM162A	CREBRF	DCN	CPXM1	PHETA1
LOXL2	COL11A1	PEAR1	PTGER4	MARS1	FAM171B	FAT3	SYT11	ALOX5	RDH11	GLMP	
HTRA1	EVI2A	CD36	CNTNAP3	CLCN5	NADSYN1	APOE	RAB20	RIMS2	GPAT3	PYGM	
DRAXIN	EEPD1	MAP3K4	SULT1E1	DOK5	CTSH	STK10	HSPH1	C14orf132	STC1	RNF41	
MAFB	SPOCK1	MOV10	CERS1	PLOD1	HERPUD1	CLEC18A	RAB31	PGPEP1	STAT4	TIMP1	
SNN	PLPP4	BDKRB2	SRPX	CPT1A	GPRIN2	KCNN3	MCF2L2	FLCN	PCDH1	HAX1	
C1QTNF5	KRT18	DNER	PGM1	LEF1	ENO1	ADARB1	ARL8B	BRI3	PSAP	RAB3IL1	
MFRP	CMTM3	RNF207	ABCA8	STING1	UTP25	POU3F2	CD276	TMEM86A	PMP22	PCDHB3	

Supplementary Figure 15. Full list of 641 DEGs common between astrocytes grown on Rq_{12}, Rq_{16}, and Rq_{24} versus smooth.

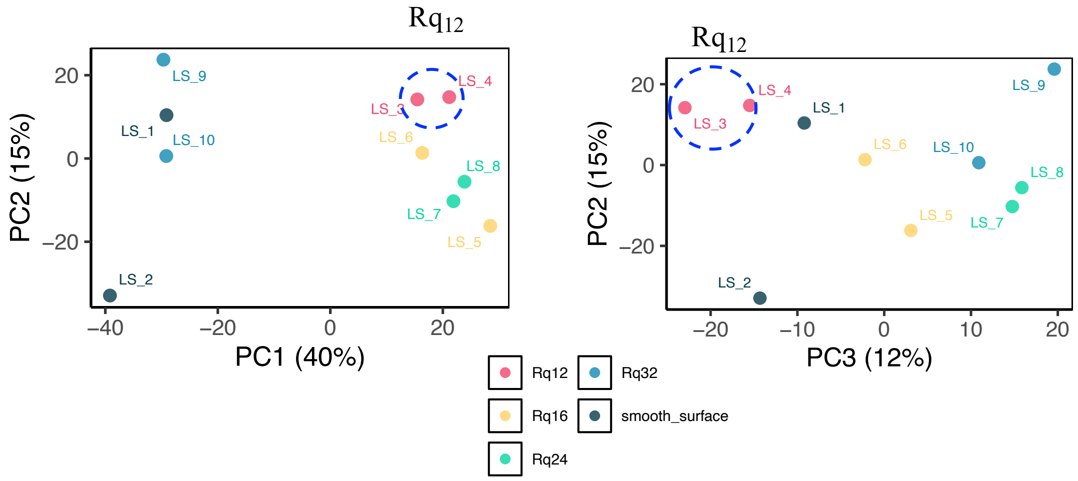

Supplementary Figure 16. Principal component analysis of the expressed genes. PCA was performed on log-transformed counts-per-million gene expression data. The top three principal components (PC1, PC2, and PC3) are shown in pairwise scatterplots, with samples colored by condition. (**Label: Smooth:** LS_1, LS_2, **Rq_{12}:** LS_3, LS_4, **Rq_{16}:** LS_5, LS_6, **Rq_{24}:** LS_7, LS_8, **Rq_{32}:** LS_9, LS_10). The percent variance explained by each PC is indicated on the axes. The PCA plot highlights the similarity between the gene expression profiles of astrocytes grown on both extremes of the nanoroughness range, $Rq_{3.5}$ and Rq_{32}, differing significantly from Rq_{12}, Rq_{16}, and Rq_{24}, and with Rq_{12} showing a unique trend.

[1] "GUENTHER_GROWTH_SPHERICAL_VS_ADHERENT_UP"
[2] "HP_GLIOBLASTOMA_MULTIFORME"
[3] "NUTT_GBM_VS_AO_GLIOMA_DN"
[4] "NUTT_GBM_VS_AO_GLIOMA_UP"
[5] "TCGA_GLIOBLASTOMA_COPY_NUMBER_UP"
[6] "VERHAAK_GLIOBLASTOMA_CLASSICAL"
[7] "VERHAAK_GLIOBLASTOMA_MESENCHYMAL"
[8] "VERHAAK_GLIOBLASTOMA_NEURAL"
[9] "VERHAAK_GLIOBLASTOMA_PRONEURAL"
[10] "WP_GLIOBLASTOMA_SIGNALING"
[11] "YAMANAKA_GLIOBLASTOMA_SURVIVAL_DN"
[12] "COLIN_PILOCYTIC_ASTROCYTOMA_VS_GLIOBLASTOMA_DN"
[13] "COLIN_PILOCYTIC_ASTROCYTOMA_VS_GLIOBLASTOMA_UP"
[14] "HP_ASTROCYTOMA"
[15] "SHARMA_ASTROCYTOMA_WITH_NF1_SYNDROM"
[16] "SHARMA_PILOCYTIC_ASTROCYTOMA_LOCATION_DN"
[17] "SHARMA_PILOCYTIC_ASTROCYTOMA_LOCATION_UP"
[18] "WP_PILOCYTIC_ASTROCYTOMA"
[19] "CAHOY_ASTROCYTIC"
[20] "DESCARTES_FETAL_CEREBELLUM_ASTROCYTES"
[21] "DESCARTES_FETAL_CEREBRUM_ASTROCYTES"
[22] "DESCARTES_FETAL_EYE_ASTROCYTES"
[23] "DESCARTES_MAIN_FETAL_ASTROCYTES"
[24] "FAN_EMBRYONIC_CTX_ASTROCYTE_1"
[25] "FAN_EMBRYONIC_CTX_ASTROCYTE_2"
[26] "GAVISH_3CA_MALIGNANT_METAPROGRAM_25_ASTROCYTES"
[27] "GOBP_ASTROCYTE_ACTIVATION"
[28] "GOBP_ASTROCYTE_CELL_MIGRATION"
[29] "GOBP_ASTROCYTE_DEVELOPMENT"
[30] "GOBP_ASTROCYTE_DIFFERENTIATION"
[31] "GOBP_GLIAL_CELL_PROLIFERATION"
[32] "GOBP_NEGATIVE_REGULATION_OF_ASTROCYTE_ACTIVATION"
[33] "GOBP_NEGATIVE_REGULATION_OF_ASTROCYTE_DIFFERENTIATION"
[34] "GOBP_POSITIVE_REGULATION_OF_ASTROCYTE_DIFFERENTIATION"
[35] "GOBP_REGULATION_OF_ASTROCYTE_ACTIVATION"
[36] "GOBP_REGULATION_OF_ASTROCYTE_DIFFERENTIATION"
[37] "GOCC_ASTROCYTE_END_FOOT"
[38] "GOCC_ASTROCYTE_PROJECTION"
[39] "GOMF_S100_PROTEIN_BINDING"

[40] "HP_ABNORMAL_ASTROCYTE_MORPHOLOGY"
[41] "HP_ASTROCYTOSIS"
[42] "LEIN_ASTROCYTE_MARKERS"
[43] "MENSE_HYPOXIA_UP"
[44] "ZHONG_PFC_C1_ASTROCYTE"
[45] "ZHONG_PFC_C2_ASCL1_POS_ASTROCYTE"
[46] "ZHONG_PFC_C3_ASTROCYTE"
[47] "ZHONG_PFC_MAJOR_TYPES_ASTROCYTES"
[48] "Reactive_astrocytes_Leng et al. (2022)"
[49] "Reactive_astrocytes_Morabito et al. (2021)"
[50] "Reactive_astrocytes_Zhou et al. (2020)"
[51] "Reactive_astrocytes_Grubman et al. (2019)"
[52] "Reactive_astrocytes_Mathys et al. (2019)"
[53] "Reactive_astrocytes_Lau et al. (2020)"
[54] "Reactive_astrocytes_Leng et al. (2021)"
[55] "Reactive_astrocytes_Sadick et al. (2022)"
[56] "Reactive_astrocytes_Serrano-Pozo et al. (2022)"
[57] "Reactive_astrocytes_Smajic et al. (2022)"
[58] "Reactive_astrocytes_Al-Dalahmah et al. (2020)"
[59] "Reactive_astrocytes_Absinta et al. (2021)"
[60] "Reactive_astrocytes_Chancellor et al. (2021)"
[61] "REACTIVE_ASTROCYTES_Matusova et al. (2023)"

Supplementary Figure 17. Gene sets included in GSEA analysis. List of 61 curated gene sets used for gene set enrichment analysis of differentially expressed genes in Rq12 astrocytes compared with all other conditions. The panel encompasses (i) glioblastoma-related signatures (e.g., Verhaak subtypes, TCGA CNV, Nutt and Colin comparisons, Yamanaka survival, WP signaling, HP terms), (ii) astrocytoma/low-grade glioma gene sets (e.g., pilocytic astrocytoma, NF1-associated astrocytoma, Cahoy astrocytic markers, embryonic and fetal astrocyte programs), and (iii) reactive astrocyte signatures derived from recent human single-cell and bulk studies (Absinta 2021; Leng 2021, 2022; Matusova 2023; Morabito 2021; Grubman 2019; Mathys 2019; Lau 2020; Sadick 2022; Serrano-Pozo 2022; Smajic 2022; Zhou 2020; Al-Dalahmah 2020; Chancellor 2021). Together, these sets provide a broad reference landscape for evaluating whether the Rq12 transcriptome engages transcriptional programs associated with glioblastoma biology, astrocytoma/low-grade glioma, or reactive astrocyte states.

	setSize	enrichmentScore	NES	pvalue	p.adjust	qvalue	rank	leading_edge
VERHAAK_GLIOBLASTOMA_PRONEURAL	149	0.4474884	1.844819	0.000006	0.000323	0.0002446255	2648	tags=27%, list=14%, signal=23%
VERHAAK_GLIOBLASTOMA_NEURAL	102	0.4799669	1.860455	0.000036	0.000901	0.0006832117	3882	tags=41%, list=21%, signal=33%
Reactive_astrocytes_Absinta et al. (2021)	56	−0.5664273	−1.910632	0.000122	0.002026	0.0015354889	816	tags=20%, list=4%, signal=19%
Reactive_astrocytes_Leng et al. (2022)	33	−0.6394388	−1.943892	0.000360	0.004499	0.0034100807	2557	tags=45%, list=14%, signal=39%
COLIN_PILOCYTIC_ASTROCYTOMA_VS_GLIOBLASTOMA_DN	27	0.6201237	1.834656	0.001405	0.014049	0.0106480191	1398	tags=22%, list=7%, signal=21%
Reactive_astrocytes_Grubman et al. (2019)	33	−0.5600587	−1.702577	0.005940	0.049502	0.0375171998	1117	tags=36%, list=6%, signal=34%
FAN_EMBRYONIC_CTX_ASTROCYTE_1	68	0.4325112	1.554655	0.008122	0.050765	0.0384748702	3262	tags=38%, list=17%, signal=32%
ZHONG_PFC_MAJOR_TYPES_ASTROCYTES	297	0.3000263	1.353521	0.007321	0.050765	0.0384748702	2480	tags=19%, list=13%, signal=17%
SHARMA_PILOCYTIC_ASTROCYTOMA_LOCATION_UP	24	0.5527275	1.601445	0.015973	0.079865	0.0605294258	2901	tags=46%, list=15%, signal=39%
REACTIVE_ASTROCYTES_Matusova et al. (2023)	254	−0.3225959	−1.373171	0.014863	0.079865	0.0605294258	1239	tags=14%, list=7%, signal=13%
GOBP_ASTROCYTE_ACTIVATION	18	−0.5777846	−1.526382	0.027493	0.109453	0.0829535506	2051	tags=28%, list=11%, signal=25%
FAN_EMBRYONIC_CTX_ASTROCYTE_2	137	0.3257258	1.325284	0.027779	0.109453	0.0829535506	2876	tags=27%, list=15%, signal=23%
VERHAAK_GLIOBLASTOMA_MESENCHYMAL	188	−0.3195178	−1.313733	0.028458	0.109453	0.0829535506	4599	tags=35%, list=25%, signal=26%
DESCARTES_MAIN_FETAL_ASTROCYTES	23	0.5121720	1.460376	0.031788	0.113527	0.0860414852	2215	tags=30%, list=12%, signal=27%
Reactive_astrocytes_Chancellor et al. (2021)	37	0.4443307	1.422688	0.059341	0.181795	0.1377811494	1125	tags=22%, list=6%, signal=20%
COLIN_PILOCYTIC_ASTROCYTOMA_VS_GLIOBLASTOMA_UP	31	0.4490303	1.388009	0.061810	0.181795	0.1377811494	2087	tags=32%, list=11%, signal=29%
CAHOY_ASTROCYTIC	76	0.3674884	1.343064	0.058140	0.181795	0.1377811494	2467	tags=25%, list=13%, signal=22%
DESCARTES_FETAL_CEREBELLUM_ASTROCYTES	68	0.3638638	1.307903	0.084091	0.228577	0.1732372952	3082	tags=32%, list=16%, signal=27%
ZHONG_PFC_C1_ASTROCYTE	28	0.4326484	1.302780	0.104213	0.228577	0.1732372952	5819	tags=54%, list=31%, signal=37%
GAVISH_3CA_MALIGNANT_METAPROGRAM_25_ASTROCYTES	49	0.3841836	1.300639	0.098131	0.228577	0.1732372952	1016	tags=22%, list=5%, signal=21%
NUTT_GBM_VS_AO_GLIOMA_DN	43	0.3917731	1.297385	0.105145	0.228577	0.1732372952	733	tags=12%, list=4%, signal=11%
MENSE_HYPOXIA_UP	97	−0.3314270	−1.248059	0.102655	0.228577	0.1732372952	5351	tags=44%, list=29%, signal=32%
ZHONG_PFC_C3_ASTROCYTE	333	−0.2688494	−1.171721	0.103834	0.228577	0.1732372952	3346	tags=27%, list=18%, signal=23%
Reactive_astrocytes_Morabito et al. (2021)	18	0.5090123	1.363599	0.124464	0.259299	0.1965213463	2252	tags=39%, list=12%, signal=34%
DESCARTES_FETAL_CEREBRUM_ASTROCYTES	76	0.3361649	1.228586	0.130233	0.260465	0.1974051408	3082	tags=33%, list=16%, signal=28%

	setSize	enrichmentScore	NES	pvalue	p.adjust	qvalue	rank	leading_edge
DESCARTES_FETAL_EYE_ASTROCYTES	23	0.4523489	1.289801	0.139535	0.268336	0.2033707	1651	tags=26%, list=9%, signal=24%
Reactive_astrocytes_Smajic et al. (2022)	29	0.4081399	1.231789	0.179204	0.331858	0.2515137	1428	tags=21%, list=8%, signal=19%
GOBP_NEGATIVE_REGULATION_OF_ASTROCYTE_DIFFERENTIATION	10	0.5449914	1.243258	0.220408	0.393586	0.2982968	3809	tags=50%, list=20%, signal=40%
GOBP_ASTROCYTE_DEVELOPMENT	32	−0.3864580	−1.169419	0.239437	0.404157	0.3063085	2051	tags=22%, list=11%, signal=20%
WP_GLIOBLASTOMA_SIGNALING	78	0.3052913	1.126586	0.242494	0.404157	0.3063085	4725	tags=35%, list=25%, signal=26%
Reactive_astrocytes_Mathys et al. (2019)	15	−0.4694347	−1.189570	0.259962	0.411740	0.3120555	3967	tags=33%, list=21%, signal=26%
LEIN_ASTROCYTE_MARKERS	33	0.3690715	1.154100	0.263514	0.411740	0.3120555	1344	tags=24%, list=7%, signal=23%
Reactive_astrocytes_Sadick et al. (2022)	14	0.4439284	1.116957	0.319588	0.484224	0.3669906	2252	tags=36%, list=12%, signal=31%
GOBP_ASTROCYTE_DIFFERENTIATION	70	0.2936029	1.065610	0.338462	0.497738	0.3772327	3962	tags=36%, list=21%, signal=28%
Reactive_astrocytes_Al−Dalahmah et al. (2020)	67	0.2926316	1.048525	0.352273	0.503247	0.3814081	2957	tags=30%, list=16%, signal=25%
GOBP_REGULATION_OF_ASTROCYTE_DIFFERENTIATION	24	0.3611909	1.046496	0.401691	0.557905	0.4228330	3809	tags=42%, list=20%, signal=33%
HP_ASTROCYTOSIS	20	−0.3780949	−1.019127	0.419173	0.566450	0.4293094	5417	tags=50%, list=29%, signal=36%
Reactive_astrocytes_Lau et al. (2020)	25	−0.3570017	−1.008637	0.446529	0.587538	0.4452922	1838	tags=20%, list=10%, signal=18%
Reactive_astrocytes_Serrano−Pozo et al. (2022)	19	0.3649578	0.988142	0.476695	0.611147	0.4631853	3189	tags=32%, list=17%, signal=26%
NUTT_GBM_VS_AO_GLIOMA_UP	45	0.2793670	0.928389	0.587699	0.734624	0.5567678	1359	tags=11%, list=7%, signal=10%
TCGA_GLIOBLASTOMA_COPY_NUMBER_UP	67	0.2532586	0.907449	0.627273	0.746753	0.5659604	6482	tags=40%, list=35%, signal=26%
HP_ABNORMAL_ASTROCYTE_MORPHOLOGY	43	−0.2816315	−0.904790	0.614414	0.746753	0.5659604	5556	tags=40%, list=30%, signal=28%
GOMF_S100_PROTEIN_BINDING	14	−0.3523105	−0.867562	0.651838	0.757951	0.5744468	2251	tags=21%, list=12%, signal=19%
GUENTHER_GROWTH_SPHERICAL_VS_ADHERENT_UP	19	−0.2998950	−0.801150	0.733962	0.834048	0.6321206	1950	tags=16%, list=10%, signal=14%
GOBP_POSITIVE_REGULATION_OF_ASTROCYTE_DIFFERENTIATION	11	−0.3217312	−0.753413	0.797710	0.886344	0.6717557	3021	tags=36%, list=16%, signal=31%
GOBP_GLIAL_CELL_PROLIFERATION	48	−0.2403230	−0.787328	0.847670	0.901777	0.6834519	3177	tags=21%, list=17%, signal=17%
HP_GLIOBLASTOMA_MULTIFORME	24	−0.2643274	−0.742269	0.843100	0.901777	0.6834519	3694	tags=38%, list=20%, signal=30%
VERHAAK_GLIOBLASTOMA_CLASSICAL	155	0.2042100	0.846178	0.877751	0.914324	0.6929610	2459	tags=14%, list=13%, signal=12%
HP_ASTROCYTOMA	24	−0.2077035	−0.583261	0.964083	0.964083	0.7306736	3694	tags=21%, list=20%, signal=17%
GOCC_ASTROCYTE_PROJECTION	14	−0.2242136	−0.552125	0.959381	0.964083	0.7306736	2164	tags=29%, list=12%, signal=25%

Supplementary Figure 18. Gene sets analyzed and the respective statistical parameters.

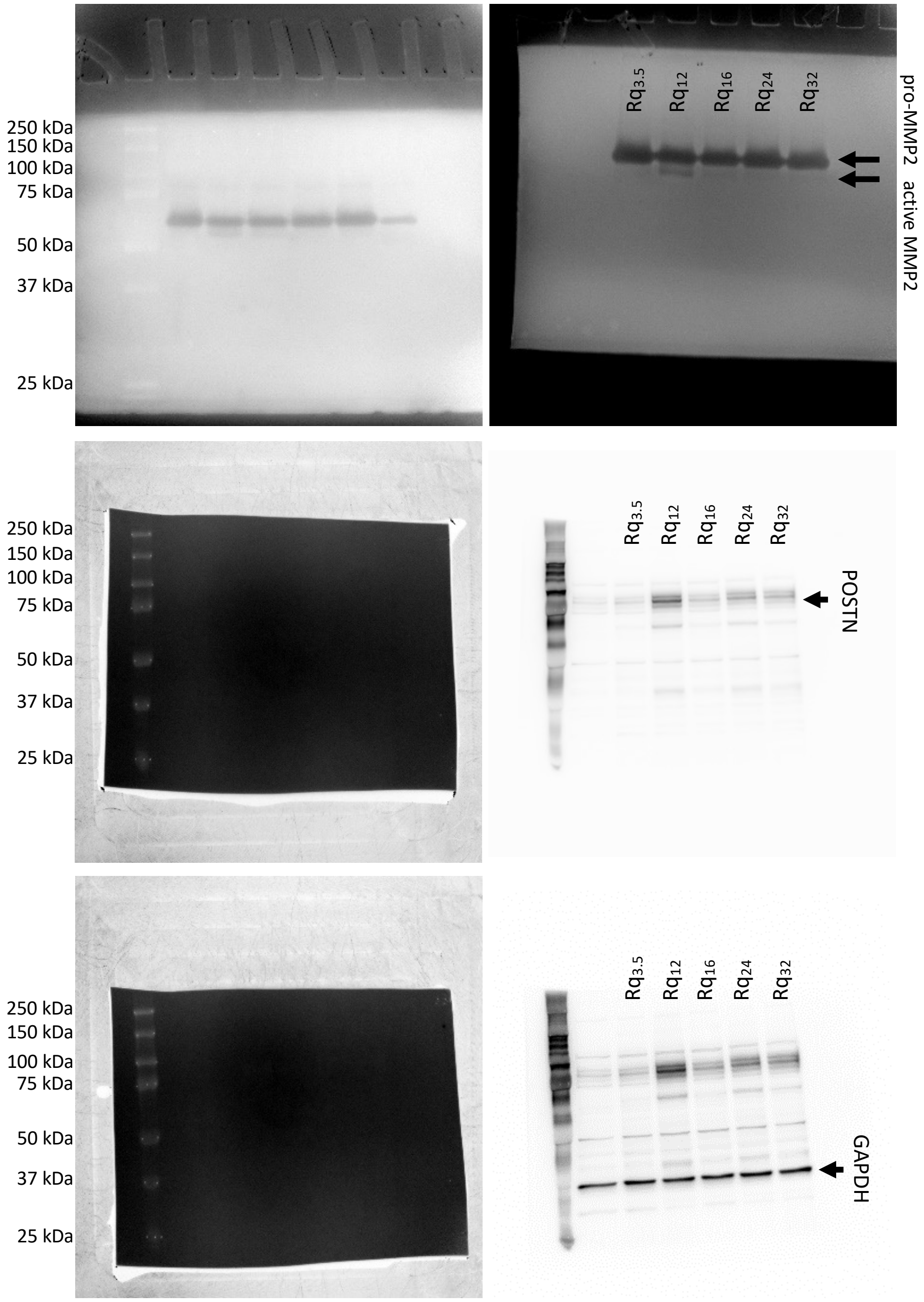

Supplementary Figure 19. Full gelatin zymography gels of MMP-2, and Western Blots of MMP2, POSTN and GAPDH shown in Figure 6d. Ladders with protein sizes indicated. MMP2: matrix metalloproteinase 2, POSTN: periostin, GAPDH: Glyceraldehyde-3-phosphate-dehydrogenase.

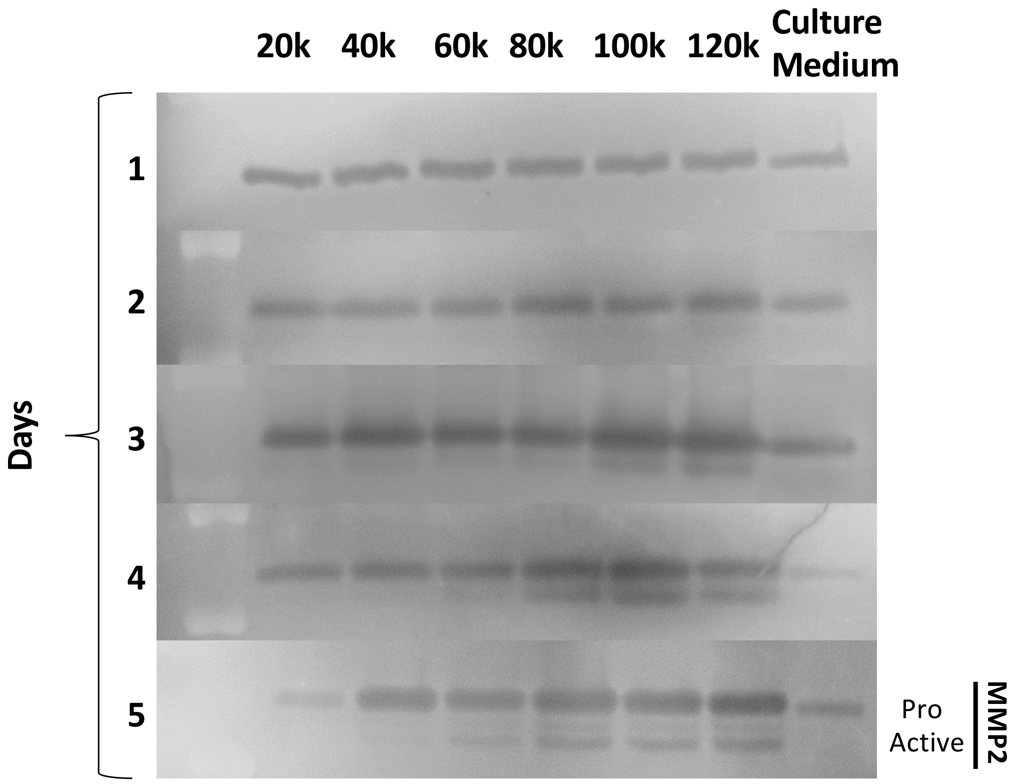

Supplementary Figure 20. Time course quantification of MMP release in the culture medium as a function of number of astrocytes seeded on Rq_{12} over 5 days by gelatin zymography. The top band at 72 kDa represents pro-MMP2, while the lower band shows active-MMP2. MMP9 is not shown as no differences were observed.

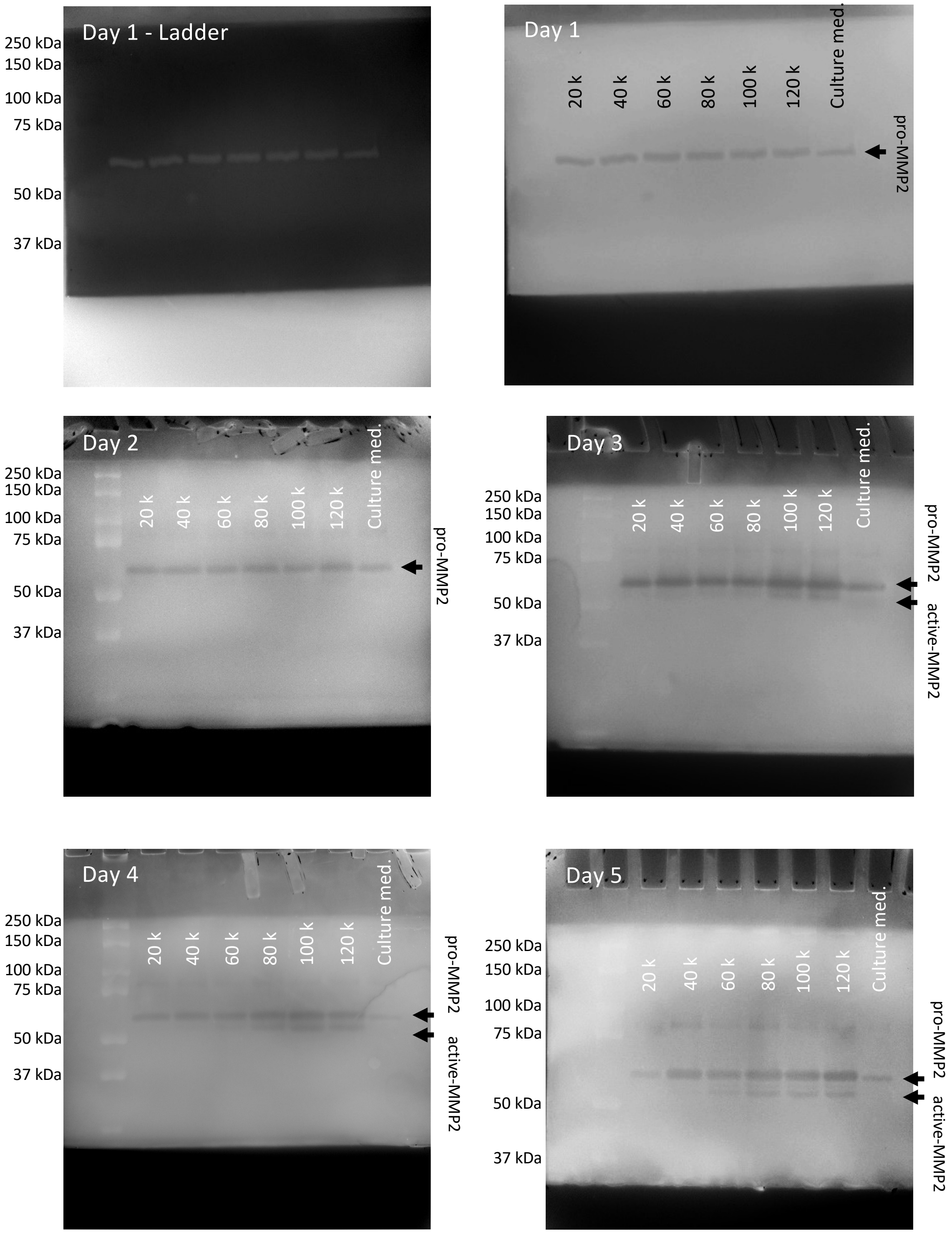

Supplementary Figure 21. Full gelatin zymography gels of matrix metalloproteinase 2 (MMP2) in Supplementary Figure 20. Ladders with protein sizes indicated.

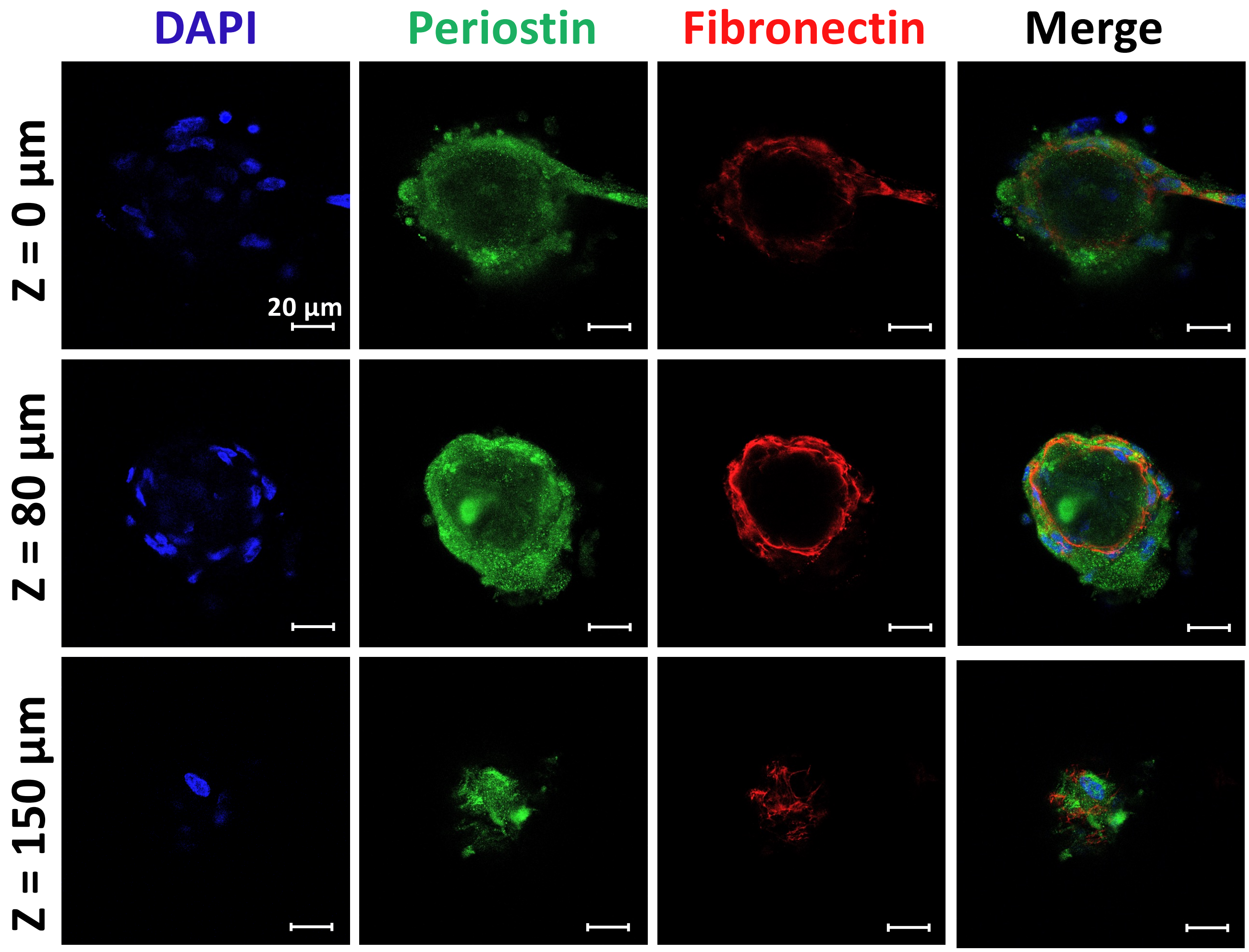

Supplementary Figure 22. Confocal imaging of an astrocyte spheroid on Rq_{12}, stained for periostin (POSTN, green), fibronectin (FN, red), and nucleus (DAPI, blue).

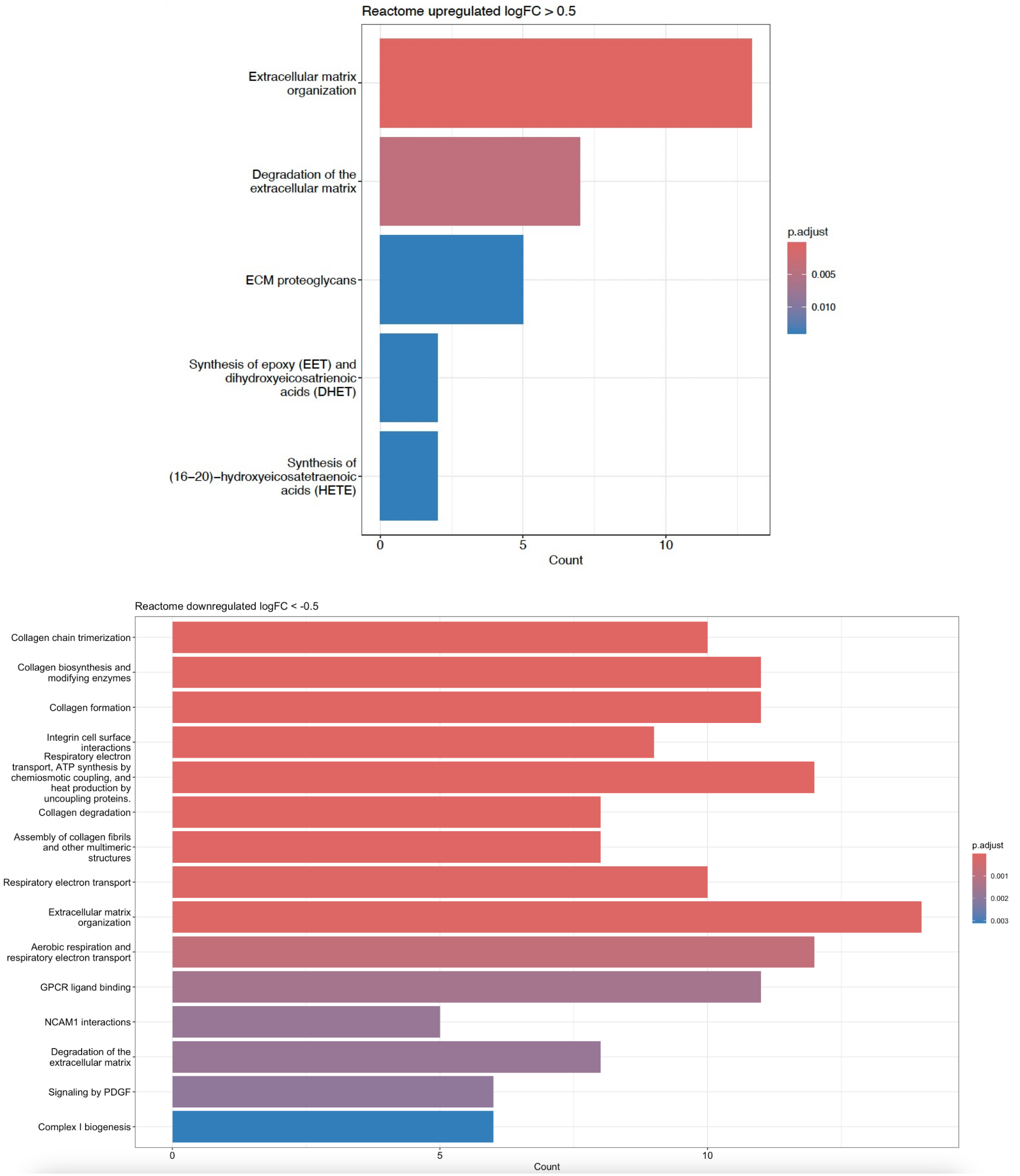

Supplementary Figure 23. Reactome pathway enrichment of upregulated (top graph) and downregulated (bottom graph) genes in Rq_{12} vs all other conditions. Bar plots showing significantly enriched Reactome pathways among genes upregulated (logFC > 0.5, adjusted p-value < 0.05) and downregulated (logFC < -0.5, adjusted p-value < 0.05) in Rq12 compared to all other conditions. Pathway enrichment was performed using the ReactomePA package. Bar lengths represent the number of upregulated (or downregulated) genes associated with each pathway, and the fill color corresponds to adjusted p-values (Benjamini-Hochberg correction).

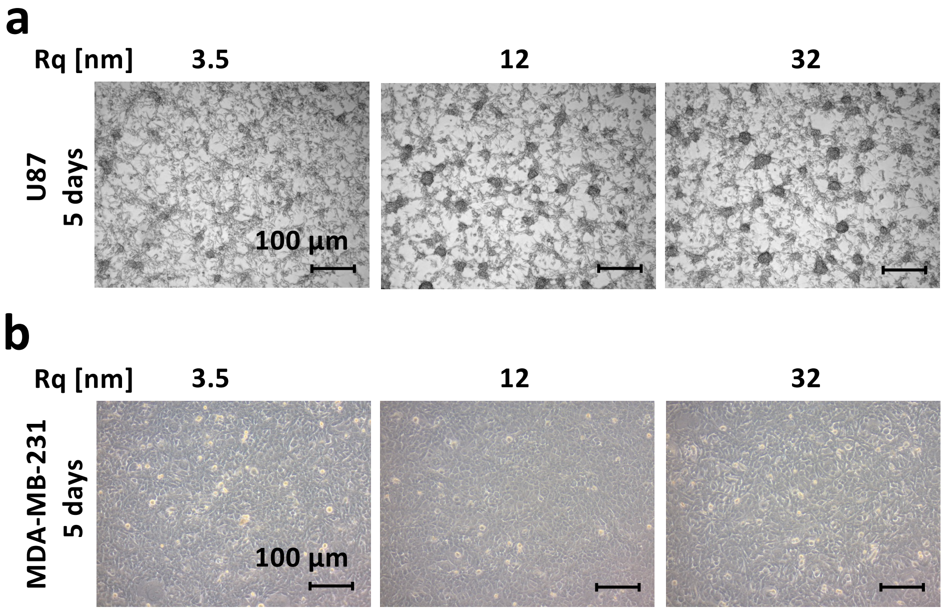

Supplementary Figure 24: **Culture of cancer cells on nanoroughness: (a)** U87 cells do not exhibit phenotypical differences between the different nanorough substrates. **(b)** MDA-MB-231 cells form a cellular monolayer regardless of the underlying nanoroughness.

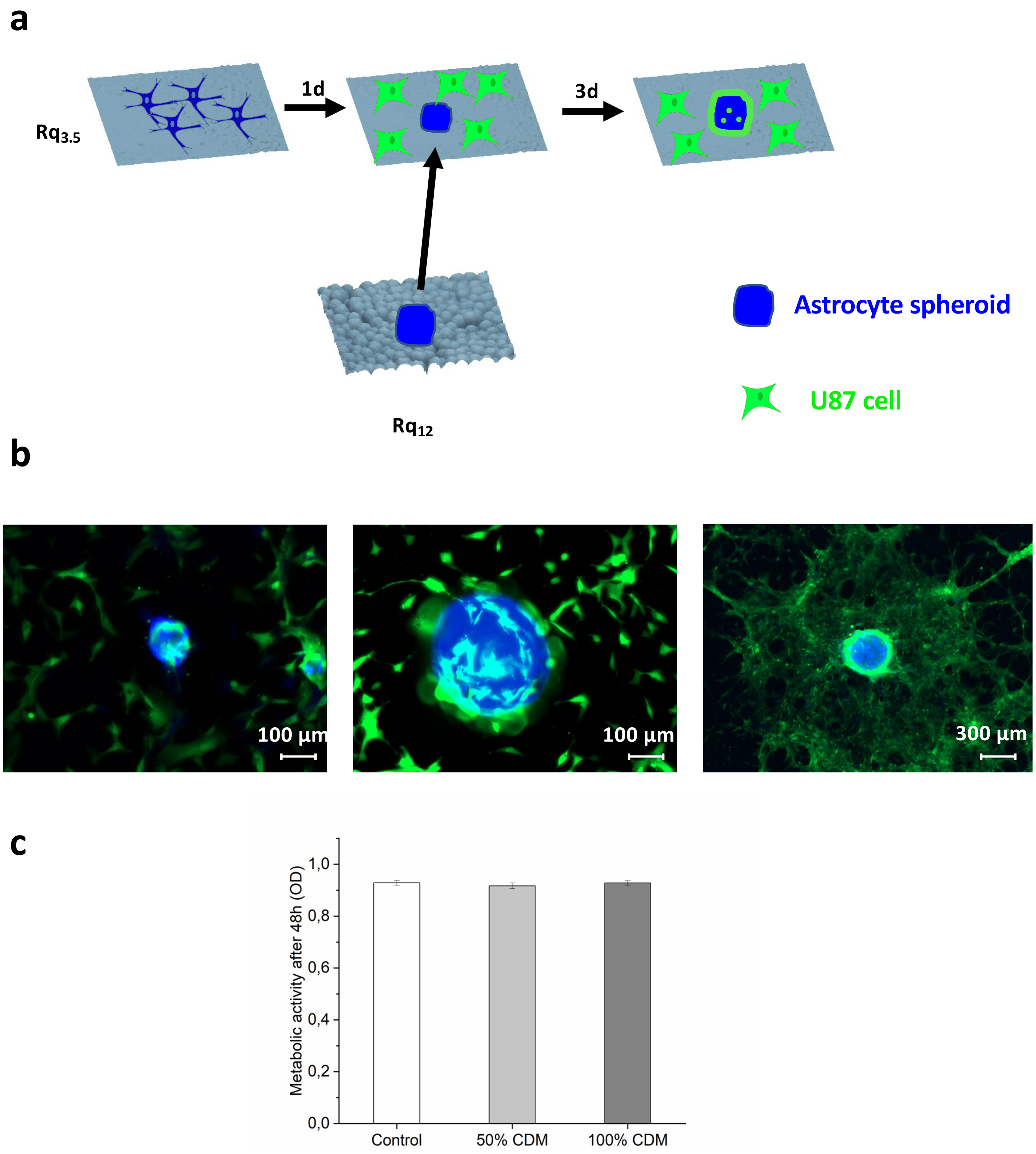

Supplementary Figure 25. Crosstalk between astrocytes and U87 cells: (a) Schematic representation of the experimental design. Astrocyte spheroids formed on Rq_{12} were trypsinized and added to U87 cultures on smooth $Rq_{3.5}$ substrate. **(b)** U87 cancer cells after 3 days of co-culture showed strong spatial association with the astrocyte spheroids. The spheroids shown the panels show were imaged in different areas of the substrate and therefore show variation in size due to nature of the samples **(c)** Assessment of metabolic activity of U87 cells after 2 days of growth in medium conditioned by astrocytes grown on Rq_{12}.

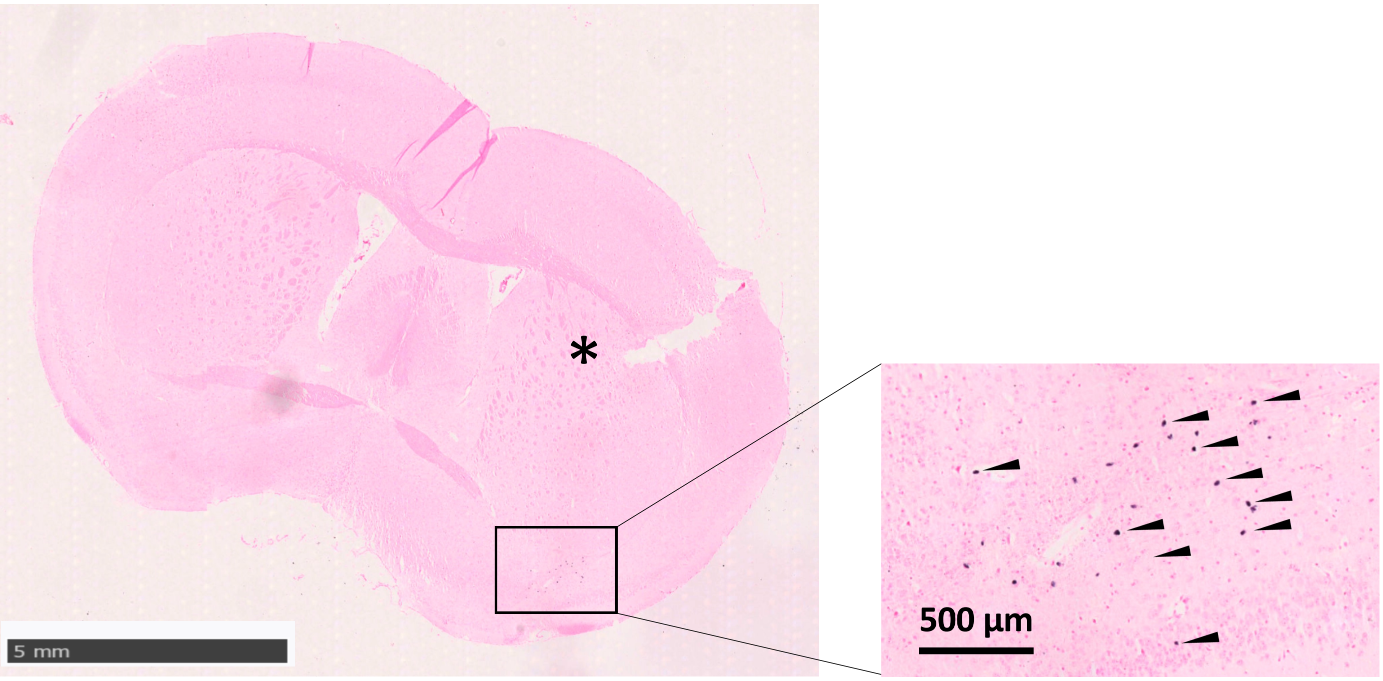

Supplementary Figure 26. Orthotopic implantation in mice: Coronal section of a representative mouse brain injected with dissociated spheroids of astrocytes grown on Rq_{12}. Human astrocytes (blue nuclei) were identified far from the injection site (indicated by the star) 12 months after injection by in situ hybridization for the Alu human sequence. The arrowheads in the higher magnification of the outlined area point toward Alu-labelled human nuclei.
